# The SLC7A11–ferroptosis axis in BRCA1-mutant breast cancer: current mechanistic evidence, potential therapeutic vulnerabilities, and translational perspectives

**DOI:** 10.3389/fonc.2026.1985224

**Published:** 2026-10-30

**Authors:** Yuying Yang, Yu Li, Xiaoyu Hao, Wenwen Yan, Xiaodong Ma

**Affiliations:** 1 College of First Clinical Medicine, Shandong University of Traditional Chinese Medicine, Jinan, China; 2 Department of Traumatic Orthopedics, The First Affiliated Hospital of Shandong Second Medical University, Weifang, China; 3 Department of Breast and Thyroid Surgery, Weifang Traditional Chinese Hospital, Weifang, China

**Keywords:** BRCA1-mutant breast cancer, disulfidptosis, ferroptosis, metabolic reprogramming, precision therapy, SLC7a11/xCT, therapeutic resistance, tumor microenvironment

## Abstract

BRCA1-mutant breast cancer is characterized by homologous recombination deficiency and genomic instability. Although these tumors are initially sensitive to platinum-based chemotherapy and PARP inhibitors, acquired treatment resistance remains a major clinical challenge. Ferroptosis is a potential therapeutic vulnerability, but the role of the SLC7A11/xCT–ferroptosis axis in this disease remains unclear. Current evidence does not establish whether increased SLC7A11 expression is a causal determinant of platinum- or PARP inhibitor resistance or a secondary response to treatment-induced stress; functional evidence directly linking SLC7A11 manipulation to drug resistance in genotype-defined BRCA1-mutant breast cancer remains limited. This review examines evidence linking SLC7A11-mediated cystine utilization to glutathione synthesis, GPX4-dependent antioxidant defense, redox and metabolic adaptation, and treatment response. We distinguish findings from BRCA1-mutant or BRCA1-deficient models from those in non-stratified breast cancer, TNBC, and broader cancer models, and discuss crosstalk with DNA damage responses and the tumor microenvironment. Individual studies have implicated VDAC3 in erastin response. BRCA1/BARD1-dependent GPX4 regulation has also been linked to ferroptosis control. However, these mechanisms require independent validation. Glucose restriction may produce overlapping but mechanistically separable ferroptotic and disulfidptotic phenotypes in SLC7A11-high cells; direct evidence for disulfidptosis in BRCA1-mutant breast cancer is lacking. Potential therapeutic strategies targeting SLC7A11, NRF2/ATF4 signaling, GPX4-related defenses, NADPH regeneration, glucose metabolism, and stromal or immune–metabolic interactions are reviewed. However, most SLC7A11-centered mechanistic and therapeutic evidence remains preclinical and has not been systematically validated in genotype-defined, platinum- or PARP inhibitor-resistant BRCA1-mutant breast cancer models. Future studies should prioritize biomarker-guided evaluation, pathway-selective experiments to distinguish ferroptosis from disulfidptosis, and prospective validation in treatment-resistant BRCA1-mutant models and clinical cohorts.

## 1 Introduction

Breast cancer remains a leading cause of cancer-related mortality in women worldwide. In 2022, approximately 2.3 million new cases and 665,000 deaths were recorded globally, underscoring its major health burden (1, 2). Among genetically defined breast cancer subgroups, breast cancer associated with pathogenic BRCA1 alterations represents a biologically distinct population characterized by impaired homologous recombination repair (HRR), genomic instability, and aggressive clinical behavior (3, 4). Pathogenic BRCA1 alterations may occur as germline or somatic mutations and can result in impaired BRCA1 function. In this review, BRCA1-mutant breast cancer refers to tumors harboring pathogenic BRCA1 alterations, whereas BRCA1 deficiency refers to the resulting functional impairment of BRCA1 activity. Large-scale prospective cohort studies have demonstrated that the cumulative risk of breast cancer by age 80 years among BRCA1 germline mutation carriers reaches 72%, with the peak annual incidence occurring between ages 41 and 50 years (28.3 per 1,000 person-years) (4). Approximately 70%–80% of BRCA1-mutant breast cancers exhibit basal-like characteristics and commonly present as triple-negative breast cancer (TNBC), whereas non-TNBC tumors, including luminal and HER2-positive subtypes, constitute a smaller proportion (5–7).

As a classic tumor suppressor gene, BRCA1 encodes a protein that primarily mediates HRR of DNA double-strand breaks and maintains genomic stability (5, 8). Based on the principle of synthetic lethality (9, 10), BRCA1-mutant tumors are initially highly sensitive to DNA-damaging therapies (11), including platinum-based chemotherapy (12) and poly(ADP-ribose) polymerase inhibitors (PARPis) (9, 12). However, acquired resistance frequently emerges during clinical treatment, resulting in therapeutic failure and poor long-term prognosis (13, 14). In addition, BRCA1 plays an important role in maintaining cellular redox homeostasis (15–18); loss of BRCA1 function induces chronic oxidative stress (15, 17, 18) and alters cellular susceptibility to ferroptotic cell death (16, 17, 19), thereby linking homologous recombination deficiency (HRD) with non-apoptotic death pathways (16, 20, 21).

Ferroptosis is an iron-dependent form of regulated cell death driven by lipid peroxidation and mechanistically distinct from apoptosis (21–23). Its regulation has been implicated in therapeutic responses and resistance across diverse cancer models (23). Beyond this broader context, emerging evidence links BRCA1 deficiency to ferroptosis-associated therapeutic vulnerability, particularly to combined GPX4 and PARP inhibition (24, 25). SLC7A11, the light-chain subunit of the cystine/glutamate antiporter system xc^−^, is a key negative regulator of ferroptosis and can influence tumor-cell sensitivity to ferroptotic stress by maintaining GSH synthesis and stabilizing redox homeostasis (26, 27). However, ferroptosis sensitivity and resistance to DNA-damaging therapy are related but distinct phenotypes, and the causal contribution of SLC7A11 to platinum or PARP inhibitor resistance in BRCA1-mutant breast cancer remains unresolved.

Although the role of SLC7A11 in ferroptosis regulation and therapeutic resistance has been extensively investigated, existing reviews mainly focus on pan-cancer mechanisms or general breast cancer contexts, with limited systematic discussion regarding the unique biological background of BRCA1-mutant breast cancer. Given that the SLC7A11–ferroptosis axis exhibits both conserved regulatory characteristics and subtype-specific features, this review aims to critically synthesize current evidence linking SLC7A11-mediated ferroptosis regulation to BRCA1-mutant breast cancer. We summarize ferroptosis-related vulnerabilities directly validated in BRCA1-mutant or BRCA1-deficient breast cancer models, carefully distinguish findings supported by direct evidence from those extrapolated from broader cancer contexts, and discuss emerging therapeutic strategies while emphasizing current preclinical limitations, unresolved resistance mechanisms, and future directions for clinical translation. By integrating BRCA1-associated homologous-recombination deficiency, genomic instability, and chronic oxidative stress with adaptation to platinum and PARP inhibitor therapy, this review frames the SLC7A11–ferroptosis axis as a context-dependent therapeutic vulnerability shaped by the redox and metabolic state of BRCA1-mutant tumors. This synthesis adds to existing pan-cancer or general breast-cancer reviews by linking BRCA1-associated biology to SLC7A11-dependent metabolic adaptation.

Literature selection. We conducted a non-systematic search of PubMed, Web of Science, and Scopus, with no predefined start date; the search was updated on 15 October 2026. Search terms included BRCA1, ferroptosis, SLC7A11, xCT/system xc^−^, GPX4, PARP inhibitor, and breast cancer. Because direct evidence in BRCA1-mutant breast cancer is limited, we also searched broader cancer literature for studies of shared redox and metabolic mechanisms. We included non-BRCA1-stratified breast cancer and non-breast cancer studies when their findings were relevant to the BRCA1-deficient context, and classified them as “Subtype-inferred” or “Extrapolated,” respectively, according to the framework in **Table 1**. Primary preclinical and clinical studies were prioritized for mechanistic and therapeutic claims; review articles were used mainly for background and conceptual context. Only English-language publications were considered.

**Table 1:** Candidate regulators of SLC7A11 implicated in ferroptosis evasion and therapeutic resistance in BRCA1-mutant breast cancer.

Candidate regulator	Regulatory class	Regulatory mechanism on SLC7A11	Relevance to BRCA1-mutant breast cancer	Evidence status	Refs.
p53	Transcription factor	Wild-type p53 binds to p53-responsive elements within the SLC7A11 promoter and represses its transcription. This mechanism has been demonstrated in breast cancer and other non-BRCA1 cancer models.	TP53 pathway alterations have been reported in BRCA-associated cancers, including ovarian cancer; however, the cited evidence does not establish a BRCA1–TP53–SLC7A11 axis in breast cancer. Loss of wild-type p53 may relieve SLC7A11 repression, but this hypothesis remains untested in genetically defined BRCA1-mutant breast cancer models.	Extrapolated	Mechanism (29, 34, 50, 69–71):; BRCA context (66–68):
BAP1	Chromatin-associated deubiquitinase and tumor suppressor	BAP1-mediated deubiquitination of histone H2A at the SLC7A11 promoter represses SLC7A11 transcription in selected non-breast cancer models.	BAP1 provides a candidate epigenetic mechanism that could influence SLC7A11 expression in BRCA1-deficient tumors; however, this pathway has not been directly evaluated in genetically defined BRCA1-mutant breast cancer models.	Extrapolated	Mechanism (30, 51):; BRCA context (40):
STAT1	Transcription factor and IFN-response regulator	STAT1-associated signaling can repress SLC7A11 expression in a context-dependent manner.	BRCA1 has been implicated in STAT1-dependent interferon responses, and BRCA1 deficiency may reduce STAT1 activity, potentially altering SLC7A11 responsiveness to inflammatory or therapeutic stress. A direct BRCA1–STAT1–SLC7A11 pathway remains unestablished in BRCA1-mutant breast cancer.	Extrapolated	Mechanism (62–65):; BRCA context (72, 73):
NRF2	Transcription factor and antioxidant-response regulator	NRF2 binds antioxidant response elements (AREs) within the SLC7A11 promoter and activates its transcription. NRF2 also coordinates glutathione biosynthesis and broader antioxidant-defense programs.	In non-BRCA1-stratified breast cancer models, NRF2 stabilization has been reported to promote SLC7A11 expression. BRCA1 has been implicated in the regulation of NRF2 stability, and BRCA1 deficiency may reduce basal NRF2 activity. Under sustained therapeutic or oxidative stress, NRF2 may be reactivated through KEAP1/p62-associated mechanisms, potentially promoting SLC7A11 upregulation. However, the temporal sequence linking BRCA1 deficiency, treatment-induced NRF2 activation, and SLC7A11 induction has not been directly validated in genetically defined BRCA1-mutant breast cancer models.	Subtype-inferred	Mechanism (52–56, 62, 74–78, 80, 81):; BRCA context (82–86, 88):
ATF4	Integrated stress-response transcription factor	ATF4 binds amino acid response elements (AAREs) within the SLC7A11 promoter and activates transcription during the integrated stress response (ISR).	In breast cancer and TNBC models without BRCA1 stratification, eIF2alpha/ATF4 signaling induces SLC7A11 and supports glutathione-dependent redox homeostasis during radiotherapy or paclitaxel treatment. Evidence from hepatocellular and colorectal cancer models further links the PERK/eIF2alpha/ATF4–SLC7A11 axis to ferroptosis resistance. This pathway therefore represents a candidate stress-response mechanism in BRCA1-mutant tumors under therapeutic pressure, although direct validation is lacking.	Subtype-inferred	Mechanism (55, 56, 77, 79, 81):
ATF3	Stress-responsive transcriptional repressor	In selected non-breast cancer models, ATF3 binds regulatory regions of the SLC7A11 promoter and represses its transcription, thereby reducing system xc^−^ activity and increasing ferroptosis sensitivity.	ATF3 represents a candidate opposing regulator of SLC7A11-mediated antioxidant defense. However, the cited studies used non-breast and non-BRCA1 models, and the relevance of ATF3 to BRCA1-mutant breast cancer remains unknown.	Extrapolated	Mechanism (57–59):
BACH1	Stress-responsive transcriptional repressor	BACH1 binds regulatory regions of the SLC7A11 promoter and represses its transcription, thereby increasing ferroptosis sensitivity in selected cancer models. This regulation can be modulated by gain-of-function mutant p53, such as p53R175H.	BACH1 represents a candidate opposing regulator of SLC7A11-mediated antioxidant defense. The mutant p53R175H–BACH1–SLC7A11 interaction has been demonstrated; however, neither the direct role of BACH1 nor its interaction with p53 has been evaluated in genetically defined BRCA1-mutant breast cancer models.	Extrapolated	Mechanism (60, 61):
cDTL	Circular RNA	Nuclear cDTL binds NRF2 to promote SLC7A11 transcription, whereas cytoplasmic cDTL binds BCLAF1 to increase SLC7A11 mRNA stability, thereby supporting the GSH–GPX4 antioxidant axis.	cDTL is a direct transcriptional target of MYC in breast cancer models, and MYC amplification is enriched in BRCA1-associated breast cancer. This provides a biological rationale for a possible MYC–cDTL–SLC7A11 pathway; however, the pathway has not been directly validated in genetically defined BRCA1-mutant breast cancer models.	Subtype-inferred	Mechanism (90):; BRCA context (91):
NORAD	Long non-coding RNA	NORAD recruits FUS to stabilize NR3C1 mRNA. NR3C1 subsequently enhances SLC7A11 transcription, forming an indirect NORAD–FUS–NR3C1–SLC7A11 regulatory cascade.	In non-BRCA1-stratified breast cancer models, NORAD promotes SLC7A11 expression and suppresses erastin-induced ferroptosis. Its relationship to BRCA1 mutation status and treatment resistance remains unknown.	Subtype-inferred	Mechanism (92):
PRSS3	Serine protease	In TNBC and other breast cancer models, PRSS3, predominantly the PRSS3-V1 isoform, interacts with PAR2 and enhances ERK1/2 signaling, leading to increased SLC7A11 and GPX4 expression and reduced ferroptosis sensitivity.	PRSS3 represents a breast cancer-associated regulatory input to the SLC7A11/GPX4 ferroptosis-defense axis. However, the reported models were not stratified by BRCA1 mutation status; whether the PRSS3–PAR2–ERK1/2–SLC7A11 pathway operates in genetically defined BRCA1-mutant breast cancer remains unknown.	Subtype-inferred	Mechanism (93):
OTUD5	Deubiquitinase	OTUD5 removes K48-linked polyubiquitin chains from SLC7A11, thereby increasing its protein stability and suppressing ferroptosis.	In non-BRCA1-stratified TNBC models, the OTUD5–SLC7A11 axis promotes tumor progression and reduces paclitaxel sensitivity through ferroptosis suppression. Its relevance to BRCA1-mutant breast cancer is inferred from TNBC overlap and requires direct validation in genetically defined BRCA1-mutant models.	Subtype-inferred	Mechanism (33):

Evidence status: “Direct” indicates evidence from BRCA1-mutant or BRCA1-deficient breast cancer models; “Subtype-inferred” indicates evidence from breast cancer models not stratified by BRCA1 status; and “Extrapolated” indicates evidence from non-breast cancer models or other contexts not directly examined in BRCA1-mutant breast cancer. The relevance of subtype-inferred and extrapolated evidence to BRCA1-mutant breast cancer remains to be validated. None of the rows currently meets the criteria for “Direct” status. The BRCA1 background of supporting models is specified, where reported, as a pathogenic mutation, experimentally induced BRCA1 deficiency, or an uncharacterized background.

## 2 SLC7A11-associated ferroptosis evasion and therapeutic adaptation

### 2.1 SLC7A11 upregulation as an adaptive response to therapeutic stress

Platinum-based agents and PARPis impose DNA damage and replication stress and can increase oxidative stress in BRCA1-mutant breast cancer cells (24, 26, 28). Although these insults promote tumor cell death, they can also elicit adaptive stress responses (26). Across cancer models, transcriptional induction of SLC7A11 has been implicated in cellular survival under oxidative and metabolic stress (26, 29, 30). Therapy-associated SLC7A11 upregulation has been reported in selected breast cancer models, particularly in TNBC cells exposed to chemotherapy (31). These observations establish that SLC7A11 expression can rise in response to therapeutic stress and that high SLC7A11 often co-occurs with resistant or aggressive phenotypes; they do not by themselves establish that SLC7A11 is necessary or sufficient for drug resistance.

In TNBC, which substantially overlaps with basal-like breast cancer, SLC7A11-mediated cystine import supports tumor-cell proliferation and redox homeostasis (32). Tissue microarray analyses further associate elevated SLC7A11 expression with adverse outcomes in TNBC (33, 34). These associations are correlative: they identify SLC7A11 as a marker of aggressive disease but do not demonstrate that SLC7A11 drives resistance. By contrast, interventional studies in TNBC have established a causal contribution of the OTUD5–SLC7A11 axis to chemotherapy resistance: OTUD5 stabilizes SLC7A11 by removing its K48-linked ubiquitin chains, and OTUD5 knockdown sensitizes TNBC cells to paclitaxel, an effect reversed by SLC7A11 overexpression (33). This loss-of-function and rescue evidence supports a causal contribution of the OTUD5–SLC7A11 axis to paclitaxel resistance in this context.

Across other breast cancer subtypes, both correlative and causal evidence exist. In Luminal A tumors, SLC7A11 sustains GSH synthesis and protects against CDK4/6 inhibitor-induced ferroptosis, whereas higher expression is associated with reduced CDK4/6 inhibitor sensitivity and shorter overall survival; mechanistic studies using knockdown, rescue, and xenograft models show that SLC7A11 is causally required for this protection (35). In ER-positive disease, SLC7A11 and SLC3A2 expression correlates with adverse prognosis, and genetic suppression of the SLC7A11/SLC3A2 axis increased endocrine-treatment sensitivity in ER-positive breast cancer models (36). In HER2-positive breast cancer, FGFR4 has been reported to induce SLC7A11 through the β-catenin/TCF4 axis, thereby suppressing ferroptosis and reducing sensitivity to anti-HER2 therapy (37); this axis was identified by genome-wide CRISPR screening and validated in patient-derived xenograft and organoid models. Independently, xCT-targeted vaccination potentiates HER2-directed immunotherapy in preclinical models (38). In trastuzumab-resistant HER2-positive models, SLC7A11 inhibition synergizes with trastuzumab to induce ferroptosis, and epigenetic remodeling contributes to SLC7A11 upregulation (39). Collectively, these interventional studies demonstrate, within their respective subtype and therapy contexts, that SLC7A11 can function as a causal mediator of treatment resistance; however, none of these studies was performed in genotype-defined BRCA1-mutant breast cancer models.

A direct genetic link between BRCA1 deficiency and SLC7A11-mediated stress adaptation is supported only by extra-mammary evidence: in a rat model of BRCA1 haploinsufficiency, loss of BRCA1 promoted ferroptosis resistance through upregulation of SLC7A11 and GPX4 *in vivo* (alongside decreased BAP1) (40). This study supports an association between BRCA1 haploinsufficiency, increased SLC7A11/GPX4 expression, and ferroptosis resistance, but does not establish that SLC7A11 is the causal mediator. Moreover, these findings were obtained in asbestos-induced mesothelioma rather than breast cancer and addressed carcinogenesis-associated ferroptosis resistance rather than chemotherapy or therapeutic drug resistance (40). Similarly, in BRCA-proficient ovarian cancer, PARP inhibition was shown to repress SLC7A11 in a p53-dependent manner. SLC7A11 knockdown enhanced olaparib-induced cytotoxicity, whereas SLC7A11 overexpression attenuated olaparib-induced ferroptosis and cytotoxicity (41). Thus, this study provides loss-of-function and gain-of-function evidence that SLC7A11 can modulate PARP inhibitor response, but it was performed in a BRCA-proficient ovarian cancer model rather than in genotype-defined BRCA1-mutant breast cancer. These observations frame SLC7A11 as a plausible redox node and candidate mediator of treatment-associated adaptation in BRCA1-mutant breast cancer. However, its causal contribution to chemotherapy or PARP inhibitor resistance in this genotype-defined context remains experimentally untested. Clinical and translational evidence investigating the role of SLC7A11 in BRCA1-mutant breast cancer remains limited. Given the redox imbalance reported in BRCA1-mutant tumors (15, 18), SLC7A11 is a biologically plausible candidate for investigation because it couples cystine uptake to glutathione synthesis (42). However, evidence that glutathione biosynthesis is a targetable dependency comes from PI3K/AKT-driven breast cancer, a distinct context (43), and whether SLC7A11 contributes to therapeutic adaptation in BRCA1-mutant breast cancer remains to be determined.

### 2.2 Putative mechanisms of SLC7A11-associated therapeutic adaptation in BRCA1-mutant breast cancer

Evidence from breast cancer and broader cancer models points to roles for SLC7A11 in antioxidant defense (44), metabolic reprogramming (26, 45), potential redox–DNA repair crosstalk (24, 25, 46, 47), and tumor–stroma or tumor–immune interactions (48). In BRCA1-mutant breast cancer, these observations provide a rationale for investigating SLC7A11 within a multifactorial and context-dependent framework of therapeutic adaptation. Accordingly, the following discussion considers its multilevel regulation and potentially interconnected roles in antioxidant defense, metabolic reprogramming, redox–DNA repair crosstalk, tumor microenvironmental interactions, and metal-dependent oxidative stress. We also discuss the potential convergence of ferroptosis and cuproptosis through glutathione-dependent redox regulation and Fe–Cu crosstalk, while noting that these interactions have not been directly established in BRCA1-mutant breast cancer. These interrelated regulatory, antioxidant, and metabolic features are summarized in **Figure 1**.

**Figure 1:**
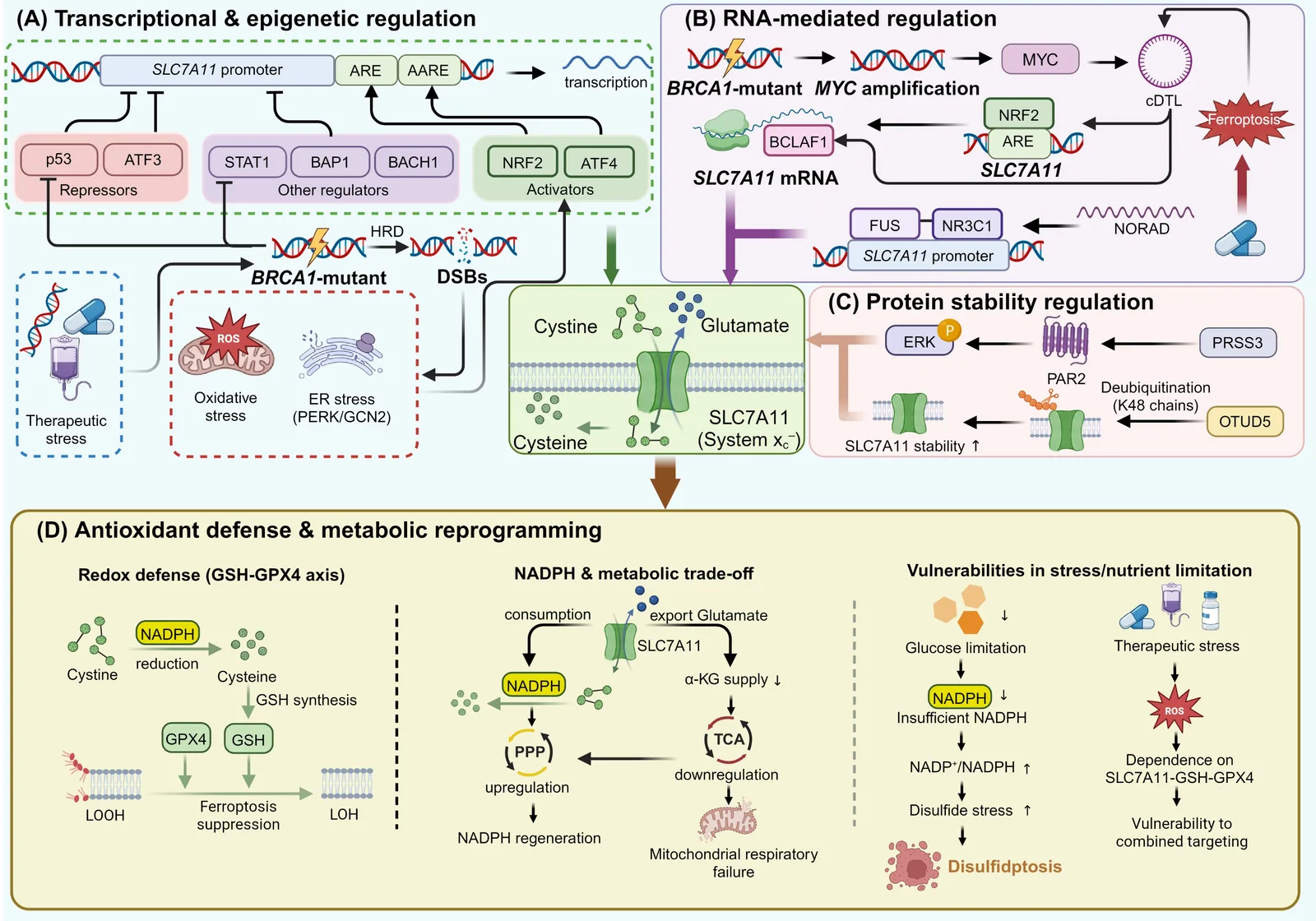
Multilevel regulation of SLC7A11 and its potential redox-metabolic roles in BRCA1-mutant breast cancer. **(A)** Transcriptional and epigenetic regulation, **(B)** RNA-mediated regulation, **(C)** protein stability regulation, and **(D)** antioxidant defense and metabolic reprogramming are depicted in separate panels. SLC7A11 expression is subject to regulation by transcriptional and epigenetic factors, including p53, ATF3, STAT1, BAP1, BACH1, NRF2, and ATF4, as well as RNA-mediated mechanisms involving cDTL and NORAD. PRSS3–PAR2–ERK signaling may further increase SLC7A11 expression, whereas OTUD5-mediated deubiquitination may enhance SLC7A11 protein stability. In BRCA1-mutant tumors, homologous recombination deficiency and associated oxidative, endoplasmic reticulum, and therapeutic stresses may influence these regulatory programs, although their contribution to SLC7A11 regulation remains incompletely defined. Through system xc^−^, SLC7A11 supports cystine uptake, glutathione synthesis, and GPX4-dependent ferroptosis defense, while potentially increasing cellular demand for NADPH regeneration and creating a metabolic constraint. Under glucose or nutrient limitation, this state may promote disulfide stress and represent a potential vulnerability to disulfidptosis or combined redox-targeting strategies. Several pathways depicted are supported by evidence from breast cancer or other cancer models and have not yet been directly validated in genetically defined BRCA1-mutant breast cancer models. Created with BioRender.com.

#### 2.2.1 Multilevel regulation of SLC7A11

SLC7A11 expression is regulated by multiple transcription factors and epigenetic regulators across breast cancer and broader cancer models (49). Within this regulatory landscape, p53 (29, 34, 50) and BAP1 (30, 51) directly repress SLC7A11 transcription, whereas NRF2 (52–54) and ATF4 (55, 56) positively regulate its expression and can promote resistance to ferroptosis. Additional transcriptional regulators, including the stress-responsive repressor ATF3 (57–59) and the context-dependent regulators BACH1 (60, 61) and STAT1 (62–65), further modulate SLC7A11 expression across stress and disease contexts. Collectively, these factors provide a candidate regulatory framework for examining how BRCA1-associated stress states may influence SLC7A11 control. The regulatory network and its evidence context are summarized in **Figure 2**, while **Table 1** details the proposed mechanisms and their relevance to BRCA1-mutant breast cancer. In this section and **Table 1**, we apply the three-tier evidence framework (“Direct,” “Subtype-inferred,” and “Extrapolated”) defined in the **Table 1** footnote. Where reported in the primary studies, the BRCA1 background is further specified as a pathogenic BRCA1 mutation, experimentally induced BRCA1 deficiency, or an uncharacterized BRCA1 background.

**Figure 2:**
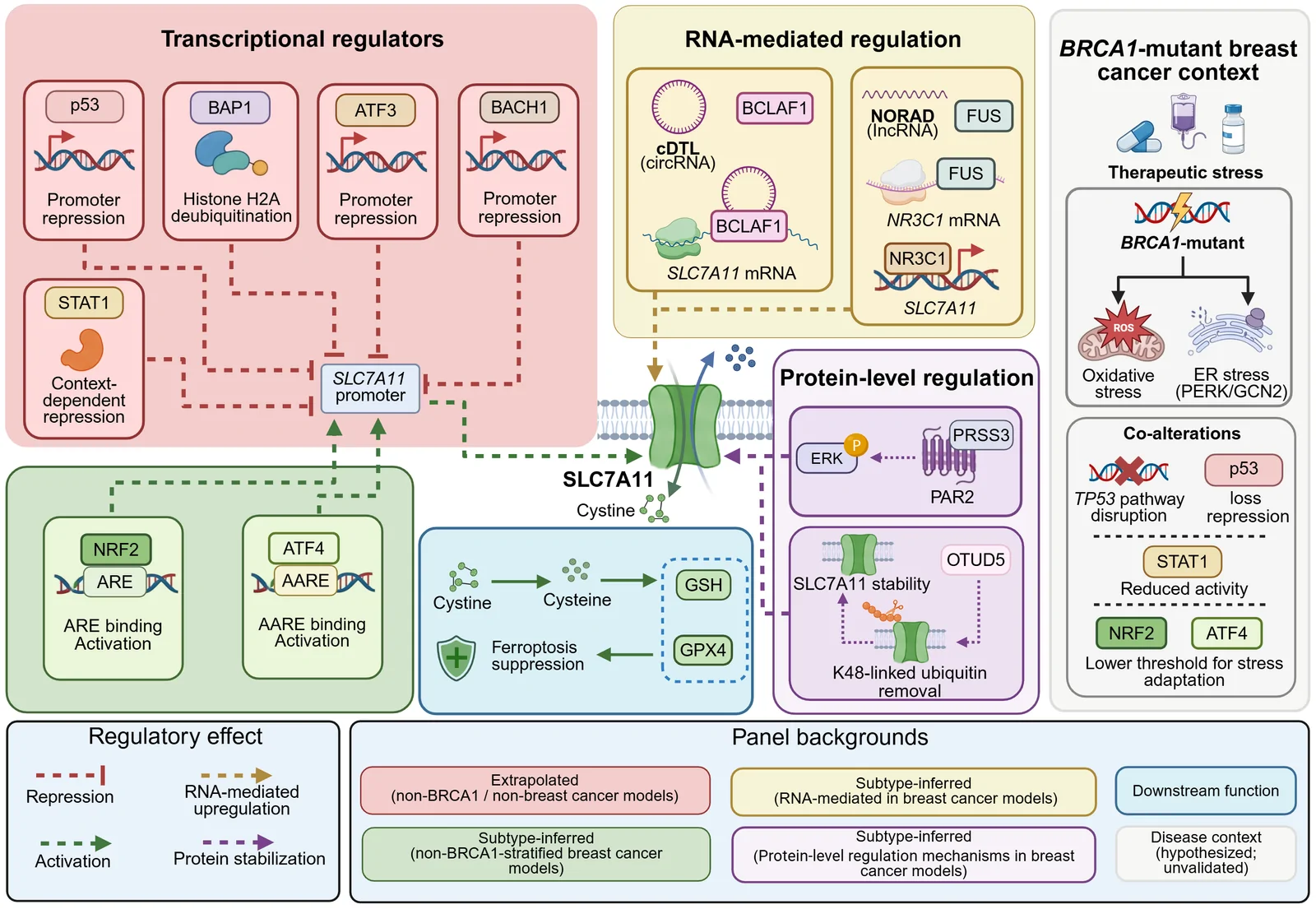
All dashed connectors within the candidate-regulator panels represent regulatory mechanisms supported by models not defined by BRCA1 mutation status, including non-BRCA1-stratified breast cancer and other cancer models, as indicated by the panel background colors. None of these mechanisms has been directly validated in genetically defined BRCA1-mutant breast cancer. Dashed blunt-ended lines denote transcriptional repression; dashed arrows denote activation (green), RNA-mediated upregulation (yellow), or protein stabilization (purple). Elements in the BRCA1-mutant breast cancer context panel represent hypothesized regulatory consequences inferred from **Table 1** (BRCA context column); the dashed connectors within this panel are likewise hypothetical and remain unvalidated in genetically defined BRCA1-mutant breast cancer models. Created with BioRender.com.

BRCA1 deficiency is associated with oxidative stress and metabolic reprogramming in BRCA1-mutant breast cancer (15–18). These stress states may increase the demand for compensatory antioxidant programs, including SLC7A11, although direct evidence for adaptive SLC7A11 upregulation following BRCA1 deficiency remains limited. Available studies in selected BRCA1-deficient models have primarily implicated GPX4- and VDAC3-related mechanisms in altered ferroptosis phenotypes (24, 25), whereas the contribution of SLC7A11 appears to vary across cellular and treatment contexts. In BRCA1-mutant tumors, additional regulatory alterations may further shape SLC7A11 responsiveness to stress. TP53 pathway disruption is frequently observed in BRCA1-associated tumorigenesis (66–68), and loss of wild-type p53 function may relieve basal repression of the SLC7A11 promoter (69–71). BRCA1 deficiency has also been associated with reduced STAT1 expression and impaired transcriptional activity (72, 73), potentially modifying the responsiveness of the SLC7A11 locus to stress-responsive regulators. Whether therapeutic stress engages SLC7A11-associated transcriptional and metabolic programs, including NRF2/ATF4 signaling, in BRCA1-mutant breast cancer warrants direct investigation.

Evidence from breast cancer and broader cancer models indicates that therapeutic oxidative stress and endoplasmic reticulum stress can induce SLC7A11 transcription through the NRF2–ARE and ISR–ATF4 pathways (55, 56, 62, 74–79). At the promoter level, NRF2 promotes SLC7A11 transcription through ARE-dependent regulatory mechanisms (74, 80), whereas ATF4 binds amino acid response elements (AAREs) to induce SLC7A11 transcription (77, 79). In TNBC models, which overlap phenotypically with but are not equivalent to BRCA1-mutant breast cancer, chemotherapy- or radiotherapy-associated stress has been linked to activation of the PERK/GCN2–eIF2α–ATF4 pathway and increased SLC7A11 expression (55, 81). An exploratory clinical study reported higher NRF2 expression in BRCA1-mutant breast tumors, suggesting that NRF2 dysregulation may occur in this molecular subgroup (82). However, mechanistic studies indicate that BRCA1 supports basal NRF2 stability and antioxidant signaling, such that BRCA1 deficiency may reduce basal NRF2 activity (83). This apparent discrepancy may reflect context-dependent NRF2 regulation: although basal NRF2 activity may be impaired after BRCA1 loss, treatment-associated oxidative or endoplasmic reticulum stress can sustain NRF2 signaling in breast cancer models, including therapy-resistant TNBC. Such stress-induced activation may involve KEAP1 sequestration or competitive KEAP1 binding by NUP62 and EDEM1 (84, 85). EGFR dysregulation in BRCA1-mutant breast cancer may provide an additional route to NRF2 activation (86, 87), although its connection to NRF2-dependent SLC7A11 regulation remains indirect. In non-BRCA1-stratified TNBC cells, EGF exposure has been associated with concurrent PI3K/AKT activation and increased NRF2 expression (88). In non-breast cancer literature, PI3K/AKT-mediated inhibition of GSK3β has been described as a mechanism that can stabilize NRF2 and facilitate expression of NRF2 target genes, including SLC7A11 (89). Against this basal regulatory background, therapeutic stress may engage an SLC7A11 locus whose responsiveness could be modified by BRCA1 deficiency, loss of p53-mediated repression, and reduced STAT1 activity. This possibility provides a mechanistic rationale for investigating NRF2/ATF4-associated SLC7A11 regulation as a candidate adaptive program in BRCA1-mutant breast cancer.

Beyond direct regulation by canonical transcription factors, SLC7A11 expression is also shaped by non-coding RNAs. In breast cancer cells, cDTL is a direct transcriptional target of MYC and enhances SLC7A11 expression through NRF2-dependent transcriptional activation in the nucleus and BCLAF1-mediated mRNA stabilization in the cytoplasm (90). MYC amplification is more frequent in BRCA1-mutant than in sporadic breast cancers (~53% vs. 23%) (91). cDTL is also induced by pharmacological ferroptotic stress and attenuates ferroptosis in breast cancer models (90). These findings raise the possibility that the MYC–cDTL–SLC7A11 axis may contribute to stress adaptation in BRCA1-mutant breast cancer. In breast cancer, the long non-coding RNA NORAD promotes SLC7A11 expression through the FUS/NR3C1 transcriptional axis, thereby attenuating erastin-induced ferroptosis (92). Although these RNA-mediated mechanisms have not been directly validated in BRCA1-mutant breast cancer, they identify candidate routes through which SLC7A11 expression and ferroptosis sensitivity may be shaped in this subtype.

Additional regulatory mechanisms include PAR2/ERK signaling and deubiquitinase-mediated control of SLC7A11 protein stability. Previous work in breast cancer models showed that PRSS3 promotes PAR2-associated ERK1/2 signaling and maintains SLC7A11 and GPX4 expression, thereby suppressing ferroptosis (93). Although this mechanism has been demonstrated in breast cancer, including TNBC models, its relevance to BRCA1-mutant breast cancer has not been directly established. Post-translational stabilization of SLC7A11 may represent an additional mechanism of adaptation to therapeutic stress. In TNBC, OTUD5 removes K48-linked polyubiquitin chains from SLC7A11, thereby stabilizing the protein, suppressing ferroptosis, and reducing paclitaxel sensitivity; OTUD5 depletion reverses these effects and enhances chemosensitivity (33). While not directly tested in BRCA1-stratified models, this TNBC evidence indicates that SLC7A11 protein stabilization can support chemotherapy tolerance. In a distinct therapeutic context, OTUD3-mediated SLC7A11 stabilization contributes to sunitinib resistance in clear cell renal cell carcinoma (94). These observations position post-translational stabilization of SLC7A11 as a plausible component of treatment-associated ferroptosis protection and a candidate adaptive mechanism for investigation in BRCA1-mutant breast cancer.

Overall, SLC7A11 regulation is best viewed as a multilayered and context-dependent process integrating basal transcriptional constraints, stress-responsive NRF2/ATF4 signaling, RNA-mediated control, and protein-stability mechanisms. In BRCA1-mutant breast cancer, BRCA1 deficiency and accompanying alterations in p53 and STAT1 may reshape the threshold at which therapeutic stress engages these regulatory layers, whereas current evidence does not support a uniform, BRCA1-loss-driven increase in SLC7A11. Accordingly, SLC7A11 represents a candidate stress-adaptive node whose functional consequences should be considered alongside the established GPX4- and VDAC3-related ferroptosis mechanisms in this subtype. This framework provides a basis for examining how *SLC7A11*-associated antioxidant defense and metabolic reprogramming, putative redox–DNA repair crosstalk, and tumor microenvironmental influences may shape therapeutic adaptation.

#### 2.2.2 SLC7A11-associated antioxidant defense and metabolic reprogramming

Building on the transcriptional framework outlined above, evidence from breast cancer and other cancer models indicates that NRF2/ATF4-dependent induction of SLC7A11 can couple treatment-associated and metabolic stress to cystine uptake, GSH biosynthesis, and amino acid adaptation, with the two factors potentially converging on SLC7A11 under selected metabolic conditions (55, 56, 62). As the light-chain subunit of system xc^−^, SLC7A11 facilitates cellular uptake of extracellular cystine. Intracellular cystine is subsequently reduced to cysteine at the expense of NADPH-derived reducing equivalents, and cysteine then serves as a rate-limiting precursor for *de novo* GSH synthesis and cellular redox maintenance (24, 26, 42, 95).

In BRCA1-mutant breast cancer, BRCA1 deficiency is associated with an elevated baseline oxidative-stress burden and metabolic dysregulation (17, 18). Therapeutic exposure may further amplify this burden. PARP inhibition increases ROS in BRCA1-deficient breast cancer cells (24). Elevated intracellular GSH has also been observed in a cisplatin-resistant MDA-MB-231 TNBC model, although its causal or dominant contribution to platinum resistance was not established (96). These observations highlight treatment-associated alterations in redox metabolism. Because SLC7A11 supplies cystine for GSH synthesis, stress-responsive induction of SLC7A11 could provide a compensatory route for sustaining GSH availability under such conditions, although this response has not been directly established in BRCA1-stratified models. Functionally, GSH serves as an essential cofactor for GPX4, a central suppressor of ferroptosis that reduces toxic phospholipid hydroperoxides to phospholipid alcohols and thereby limits the propagation of lipid peroxidation (97–99). In BRCA1-deficient cancer models, loss of BRCA1/BARD1-mediated K6-linked polyubiquitination and proteasomal degradation of GPX4 results in GPX4 protein accumulation and ferroptosis resistance (25). This BRCA1/BARD1–GPX4 axis was demonstrated primarily in ovarian cancer models, with limited additional validation in a triple-negative breast cancer cell line. Because it has not been independently reproduced in additional genotype-defined BRCA1-mutant breast cancer models, we regard it as a proposed, context-specific determinant of ferroptosis resistance rather than an established universal mechanism. Studies in BRCA1-deficient models provide stronger functional evidence for the downstream ferroptosis-protection pathway than for SLC7A11 itself. Inhibition of GPX4 can resensitize BRCA1-deficient cells and PARP inhibitor-resistant BRCA1-mutant PDX models to PARP inhibition, indicating that ferroptosis protection is functionally relevant to PARP inhibitor tolerance. Nevertheless, these studies do not demonstrate that SLC7A11 is the initiating or dominant upstream determinant of the phenotype, because GPX4 can be regulated independently of SLC7A11. Accordingly, baseline redox imbalance combined with therapy-associated transcriptional remodeling may reinforce ferroptosis protection through the SLC7A11–GSH–GPX4 axis (19, 100), providing a candidate biochemical mechanism that may contribute to treatment-associated oxidative-stress tolerance. Whether this pathway initiates or maintains platinum or PARP inhibitor resistance in BRCA1-mutant breast cancer remains to be determined.

Previous studies have described a metabolic trade-off in breast cancer, whereby antioxidant protection against ferroptosis is accompanied by increased reliance on glutamine utilization and NADPH-regenerating pathways (101, 102). Consequently, persistent or treatment-induced SLC7A11 activity may increase dependence on NADPH-regenerating pathways, particularly the pentose phosphate pathway (PPP) (42, 95). This adaptation can enhance antioxidant buffering in high-SLC7A11-expressing cells and support ferroptosis resistance, but it may also create a metabolic liability. Evidence from TNBC and other cancer models has shown that SLC7A11-high cells are particularly vulnerable to glucose limitation, under which insufficient NADPH regeneration leads to an increased NADP^+^/NADPH ratio, cystine-associated disulfide stress, and ultimately redox collapse (95, 103, 104). Collectively, these findings provide a mechanistic basis for considering PPP disruption and related NADPH-regenerating pathways as potential vulnerabilities in SLC7A11-high tumors.

Beyond its effects on NADPH homeostasis, SLC7A11-mediated glutamate export has been shown in breast cancer models to reduce intracellular glutamate availability, constrain α-ketoglutarate-supported TCA-cycle anaplerosis, and impair mitochondrial respiration and metabolic flexibility, with broader support from pan-cancer cell-line analyses (105). This constraint may be particularly consequential in BRCA1-mutant tumors, where the baseline oxidative-stress burden and metabolic dysregulation associated with BRCA1 deficiency (16–19) could increase reliance on cystine uptake and GSH-dependent redox buffering (19). However, the specific NADPH-dependence axis was not directly examined. Although this metabolic model was primarily established in non-breast cancer systems (45, 95), it provides a testable framework for investigating whether SLC7A11 activity creates a treatment-associated tradeoff between enhanced antioxidant defense and reduced metabolic flexibility in BRCA1-mutant breast cancer.

A related metabolic vulnerability is illustrated by disulfidptosis, the recently described form of regulated cell death that affects SLC7A11-high cells under glucose-limited conditions. Sustained cystine uptake and its reduction to cysteine consume NADPH and can lead to intracellular disulfide accumulation, abnormal disulfide crosslinking of actin cytoskeletal proteins, and cytoskeletal collapse (106, 107). Such glucose-limited conditions may be particularly relevant to BRCA1-mutant breast cancer, in which aberrant tumor vascularization can create regions of local nutrient deprivation (108) and BRCA1-deficient cells have been reported to rely more heavily on glucose availability (18, 109). These features provide a rationale for investigating whether SLC7A11-high cells in BRCA1-mutant breast cancer are susceptible to disulfidptosis under glucose-limited or treatment-associated metabolic stress.

Collectively, the available evidence supports a model in which SLC7A11-associated cystine utilization could contribute to GSH–GPX4-mediated ferroptosis protection in BRCA1-mutant breast cancer, while imposing additional NADPH and metabolic demands that may become important under nutrient-limited or treatment-associated conditions. This redox-metabolic state may intersect with compensatory DNA repair pathways and be further shaped by tumor microenvironmental factors, including nutrient availability and stromal support.

#### 2.2.3 Putative redox–DNA repair crosstalk

In BRCA1-mutant breast cancer and other HRD models, impaired homologous recombination creates dependence on POLQ-mediated backup repair pathways, including theta-mediated end joining (TMEJ) and microhomology-mediated gap sealing (MMGS) of post-replicative single-stranded DNA gaps (110, 111). POLQ is frequently overexpressed in HRD cancers, and its depletion is synthetically lethal with BRCA1/2 deficiency (112). Because platinum agents and PARP inhibitors impose substantial replication-associated DNA damage, POLQ-dependent backup repair may become particularly important under therapeutic pressure, providing a rationale for examining whether treatment-associated redox stress further modulates POLQ activity or stability.

Across breast cancer and other cancer models, oxidative-stress-responsive SLC7A11 induction can support intracellular GSH maintenance (26). Beyond its role in GSH–GPX4-dependent reduction of lipid peroxides across cancer models (113), GSH can regulate the activity of the DNA repair protein APE1 through reversible S-glutathionylation, as demonstrated in a cervical cancer cell model and in biochemical assays (114). Studies across diverse cancer models have suggested coordinated regulation of SLC7A11 and DNA damage-response pathways, although this relationship has not been directly characterized in BRCA1-mutant breast cancer. In esophageal squamous cell carcinoma, STC2–PRMT5 signaling promotes HR/NHEJ repair while activating the ATF4–SLC7A11/GPX4 ferroptosis-defense axis (46). In bladder cancer, IMP2 stabilizes IPO4 and SLC7A11 mRNAs, coupling PRKDC-associated DNA repair to ferroptosis suppression and cisplatin resistance (115).

Collectively, these findings support a working model in which SLC7A11-associated redox maintenance could cooperate with alternative DNA repair in HR-deficient tumors, including BRCA1-mutant breast cancer, although this interaction remains to be directly established. Accordingly, SLC7A11 or its downstream regulatory nodes may represent potential targets for disrupting the coordinated redox and DNA repair adaptations that contribute to therapeutic tolerance during platinum chemotherapy or PARP inhibitor exposure. This possibility requires direct validation using SLC7A11 loss-of-function, rescue, and drug-response experiments in genotype-defined BRCA1-mutant models.

#### 2.2.4 Putative tumor microenvironmental influences on SLC7A11-associated ferroptosis resistance

Across diverse cancer contexts, including breast cancer models, the TME provides extrinsic signals that can modulate SLC7A11 expression, ferroptosis sensitivity, and therapeutic response (48). In pancreatic ductal adenocarcinoma models, CAF-derived cysteine sustains GSH-dependent redox defense despite pharmacological SLC7A11 inhibition, thereby reducing sensitivity to ferroptosis-inducing agents (116). Demonstrated in pancreatic cancer, this stromal cysteine-support mechanism warrants investigation in BRCA1-mutant breast cancer.

CAFs and other stromal cells have been implicated in both early and established stages of BRCA1-associated breast tumorigenesis. In preneoplastic breast tissue from BRCA1 mutation carriers, MMP3-high fibroblasts acquire a pre-CAF phenotype that promotes epithelial expansion and tumor initiation (117). In established BRCA1-mutant TNBC, inflammatory CAFs promote CXCL- and VEGF-dependent angiogenesis and are associated with poorer responses to bevacizumab-containing neoadjuvant therapy (118). In a related BRCA1-IRIS-overexpressing TNBC model, tumor-derived IL-6 recruits mesenchymal stromal cells and promotes their differentiation into PGE2-secreting CAFs, establishing a feedback loop that enhances tumor growth (119). Together with the CAF-derived metabolic support described in other cancer models, these findings define a BRCA1-associated stromal context in which analogous metabolic and signaling interactions could modulate SLC7A11-associated ferroptosis resistance and therapeutic response.

Beyond nutrient provision and cytokine-mediated stromal interactions, PAR2-dependent signaling represents another potential microenvironmental input into the SLC7A11/GPX4 axis. Previous work has shown that, in breast cancer, PRSS3 overexpression is associated with adverse clinicopathological features and activates PAR2–ERK1/2 signaling to upregulate SLC7A11/GPX4, suppress ferroptosis, and reduce chemotherapy sensitivity (93). Importantly, PAR2 is not restricted to tumor cells but is also expressed by CAFs, supporting its broader role in tumor–stroma communication. In pancreatic cancer, mast cell-driven PAR2/TGF-β signaling reduces sensitivity to gemcitabine/nab-paclitaxel (120), whereas in oral squamous cell carcinoma, matriptase activates PAR2 on CAFs to promote fibroblast recruitment and aggressive disease (121). The enrichment of inflammatory CAFs in HRD-associated TNBC provides a relevant stromal context for investigating PAR2-associated ferroptosis regulation in BRCA1-mutant breast cancer (122). Coagulation factor Xa can also activate PAR2 and induce neutrophil extracellular trap formation (123). BRCA1-mutant breast tumors have been reported to exhibit inflammatory microenvironmental features (124). In this setting, inflammatory mediators may provide additional inputs to PAR2-centered tumor–stroma signaling. Collectively, these findings suggest that PAR2-centered communication between tumor cells, CAFs, and inflammatory components may represent a potential microenvironmental mechanism influencing SLC7A11/GPX4-associated ferroptosis resistance in BRCA1-mutant breast cancer.

Beyond PAR2-associated signaling, hypoxia represents another potential microenvironmental input into SLC7A11-associated ferroptosis resistance. Across solid-tumor models, hypoxia-induced HIF-1α signaling can modulate system xc⁻/SLC7A11-associated ferroptosis responses, in part through SLC1A1-mediated support of cystine uptake (125). HIF-1α expression has also been reported to be more frequently elevated in BRCA1-mutant than in sporadic breast cancer (126). Together, these observations provide a rationale for investigating whether hypoxia-associated HIF-1α signaling contributes to SLC7A11-associated ferroptosis resistance in BRCA1-mutant breast cancer.

Collectively, stromal cysteine supply, CAF-associated signaling, PAR2-dependent communication, and hypoxia may provide convergent microenvironmental inputs that could modulate SLC7A11-associated ferroptosis resistance and therapeutic response in BRCA1-mutant breast cancer. These microenvironmental inputs are summarized in **Figure 3**.

**Figure 3:**
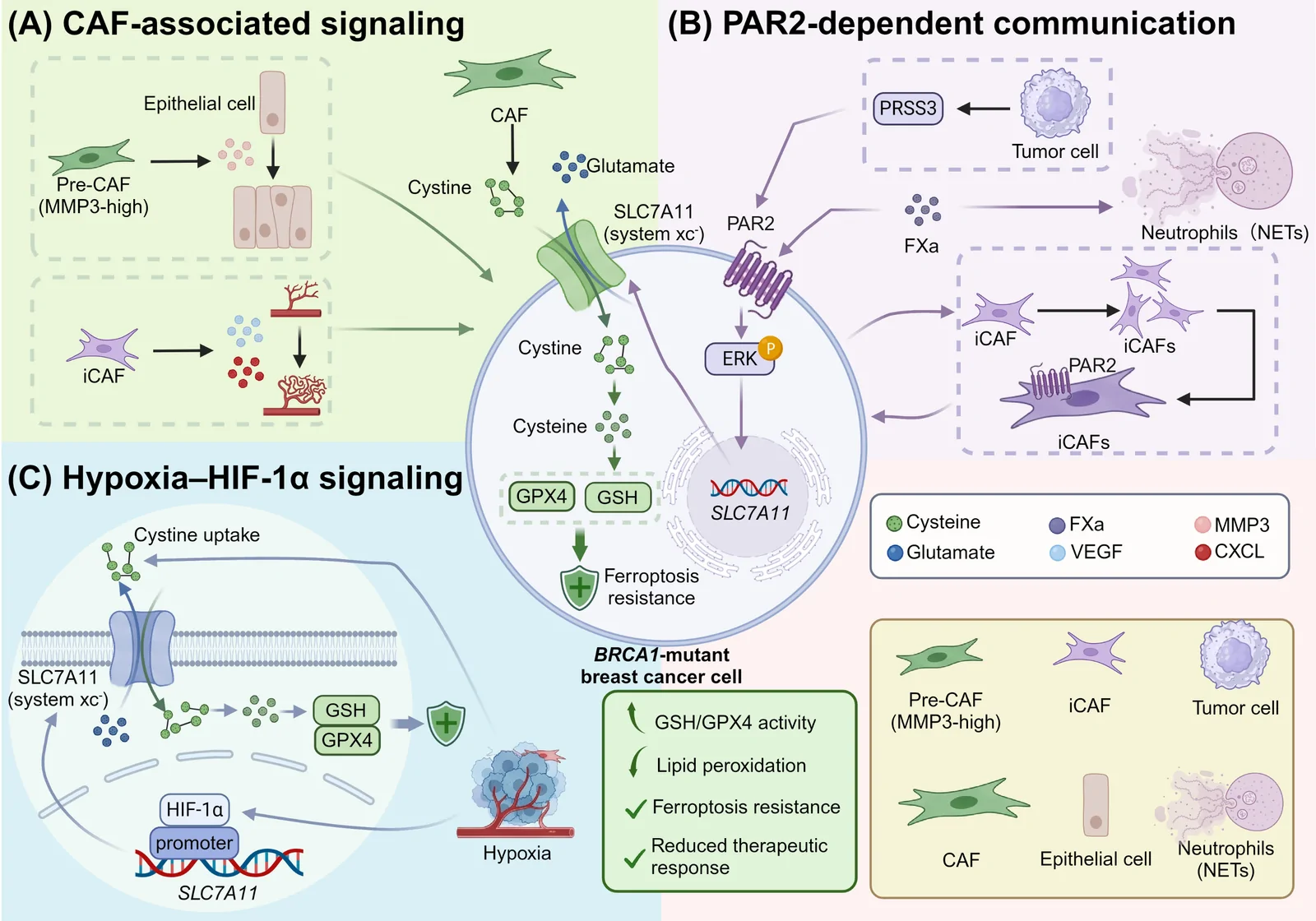
Proposed tumor microenvironmental inputs contributing to SLC7A11-associated ferroptosis resistance in BRCA1-mutant breast cancer. **(A)** MMP3-high pre-CAFs and inflammatory CAFs may support tumor initiation and angiogenic signaling and may contribute to cysteine availability, potentially sustaining tumor-cell GSH/GPX4-dependent redox defense. **(B)** Tumor-cell PRSS3 may activate PAR2–ERK signaling and increase SLC7A11 expression, whereas PAR2-positive CAFs, FXa-associated inflammatory signaling, and NET formation may provide additional tumor–stroma inputs. **(C)** Hypoxia-induced HIF-1α signaling may promote SLC7A11/system xc^−^-dependent cystine uptake and ferroptosis defense. These pathways may reduce lipid peroxidation, increase ferroptosis resistance, and impair therapeutic response. Several mechanisms are supported by evidence from breast cancer and/or other cancer models but have not been directly validated in genetically defined BRCA1-mutant breast cancer models. Created with BioRender.com.

#### 2.2.5 Metal-dependent oxidative stress and Fe–Cu crosstalk

Ferroptosis should not be considered in isolation from other metal-dependent oxidative toxicity. Although ferroptosis is primarily driven by iron-dependent lipid peroxidation of polyunsaturated fatty-acid phospholipids (21–23), copper can engage a distinct but convergent death program known as cuproptosis. Cuproptosis is triggered by copper binding to lipoylated tricarboxylic acid cycle proteins, resulting in lipoylated-protein aggregation, loss of iron–sulfur-cluster proteins, and FDX1-dependent proteotoxic stress (127). The two pathways converge on shared redox and metabolic nodes, particularly glutathione, which serves as the electron donor for GPX4 in ferroptosis and as an endogenous Cu^+^ chelator in cuproptosis (128). This dual role places glutathione at the center of the convergence: impaired glutathione redox homeostasis can amplify both ferroptotic and cuproptotic death (129), and glutathione depletion following SLC7A11 inhibition may weaken lipid-peroxide detoxification while reducing the buffering of redox-active copper (128, 129). In the tumor microenvironment, inflammation, altered metal transport, and glutathione availability may influence the iron–copper balance, consistent with copper functioning as a signaling metal in cancer (128, 130).

A self-amplifying Fe–Cu feedback loop can be envisaged. Oxidative stress and lipid peroxidation cause ATP depletion that compromises ATP7A-mediated copper export (128, 131), allowing copper to accumulate and worsen FDX1- and lipoylated-protein-dependent mitochondrial injury (127, 128). Conversely, copper deficiency or chelation may inactivate multicopper ferroxidases, including hephaestin, thereby impairing ferroportin-dependent iron efflux (132, 133), trapping iron intracellularly and enhancing Fenton-driven lipid peroxidation (128, 134). At the organismal level, loss of ferroxidase activity can produce an iron-restricted phenotype accompanied by increased transferrin receptor-mediated iron uptake (135).

In BRCA1-deficient tumors, this framework is plausible but only partly established. BRCA1 can directly regulate ferroptosis by promoting VDAC3 and GPX4 transcription; consequently, BRCA1 loss confers resistance to erastin-induced ferroptosis while increasing sensitivity to GPX4 inhibitors (24). In BRCA1-deficient cells, NCOA4-mediated ferritinophagy can promote ferroptosis during combined PARP and GPX4 inhibition (24). In a separate mechanism, BRCA1 promotes K6-linked polyubiquitination and degradation of GPX4; consequently, BRCA1 loss increases GPX4 protein abundance and confers ferroptosis resistance (25). As noted above, the generality of this mechanism in genotype-defined BRCA1-mutant breast cancer remains to be established. Together, these findings indicate that the ferroptosis phenotype associated with BRCA1 deficiency is context dependent (24, 25). Whether the SLC7A11–GSH–GPX4 axis additionally buffers redox-active copper, or whether the Fe–Cu feedback loop operates in BRCA1-mutant breast cancer, remains untested. Nevertheless, the systemic blood Zn/Cu ratio has been associated with cancer risk among BRCA1 mutation carriers (136). Resolving this will require measuring labile Fe/Cu, ATP7A/FDX1, lipoylated-DLAT oligomerization, iron–sulfur proteins, GPX4 activity, NADP+/NADPH and lipid peroxidation in genotype-defined models.

## 3 Targeting the SLC7A11–ferroptosis axis after conventional-treatment resistance

BRCA1-mutant breast cancers are commonly treated with platinum-based chemotherapy, PARP inhibitors, and other cytotoxic or targeted regimens (137), but treatment resistance frequently develops through restoration of homologous recombination (137), replication-stress adaptation (138), and redox remodeling (24). These changes may create therapeutic vulnerabilities that are not adequately addressed by continued inhibition of the original DNA-repair pathway (24). This section evaluates direct SLC7A11 inhibition, modulation of upstream regulators, and exploitation of downstream metabolic dependencies, while also considering the pharmacological, metabolic, and tumor-microenvironmental barriers that may limit *in vivo* efficacy. Targeted delivery and combination approaches are considered where they may improve drug exposure, enhance ferroptotic activity, or overcome resistance-associated limitations. Because most available evidence is derived from non-BRCA1-stratified models, the clinical relevance of these strategies remains to be established in genotype-defined, treatment-resistant systems. Candidate therapeutic strategies targeting the SLC7A11–ferroptosis axis are summarized in **Table 2** and **Figure 4**. As in **Table 1**, each row is assigned an Evidence status according to the three-tier evidence framework (“Direct,” “Subtype-inferred,” and “Extrapolated”) defined in the **Table 1** footnote, and the Supporting model/context column lists the models on which each assignment is based. Where reported in the primary studies, the BRCA1 background of supporting models is specified as a pathogenic BRCA1 mutation, experimentally induced BRCA1 deficiency, or an uncharacterized background; models for which the BRCA1 background was not reported are designated as uncharacterized.

**Table 2:** Candidate therapeutic strategies targeting the SLC7A11–ferroptosis axis with potential relevance to treatment-resistant BRCA1-mutant breast cancer.

Therapeutic strategy	Target/mechanism	Supporting model/context	Candidate biomarkers and pharmacodynamic readouts	Translational limitation	Key research gap	Evidence status	Refs.
Erastin/IKE	Inhibit system xc^−^ by targeting SLC7A11, thereby reducing cystine uptake, depleting GSH, and increasing lipid peroxidation. In BRCA1-deficient models, response to erastin-based inducers may be influenced by VDAC3 abundance.	BRCA1-deficient cancer models; ER-positive and HER2-positive breast cancer models; breast cancer nanodelivery models	SLC7A11, VDAC3, GPX4, GSH, lipid peroxidation, ROS	Reduced VDAC3 abundance may limit the activity of VDAC3-dependent erastin-based inducers. Erastin has unfavorable pharmacological properties, whereas IKE remains an experimental compound requiring further pharmacokinetic and safety evaluation.	Determine whether BRCA1 status and VDAC3 abundance predict differential sensitivity to erastin and IKE in platinum- or PARP inhibitor-resistant models.	Subtype-inferred	(23, 24, 35, 36, 144–147, 149–155)
Sorafenib	Multikinase inhibition with reported effects on system xc^−^/SLC7A11-associated ferroptosis, together with RAF-pathway modulation and anti-angiogenic activity.	ER-positive and TNBC breast cancer models, as well as hepatocellular carcinoma models	SLC7A11, GPX4, lipid peroxidation, ROS, RAF/MEK/ERK activity, and angiogenesis-related markers	Pleiotropic kinase and anti-angiogenic effects complicate attribution of antitumor activity specifically to SLC7A11 inhibition or ferroptosis. Clinically relevant ferroptotic activity in ER-positive breast cancer remains uncertain.	Separate kinase-dependent, anti-angiogenic, and ferroptosis-dependent effects and determine whether VDAC3 abundance influences sorafenib response in genetically defined BRCA1-deficient models.	Subtype-inferred	(24, 35, 36, 156, 158, 163–166)
Sulfasalazine	Inhibits system xc^−^/SLC7A11, thereby reducing cystine uptake and intracellular GSH availability.	TNBC and ER-positive breast cancer models; platinum-sensitization models	SLC7A11, GSH, ROS, cystine uptake, and lipid peroxidation	Relatively low potency, limited bioavailability, and uncertain intratumoral exposure may restrict clinical translation.	Establish pharmacodynamic SLC7A11 target engagement and evaluate combination activity with PARP inhibitors or platinum agents in treatment-resistant BRCA1-mutant models.	Subtype-inferred	(23, 31, 48, 166–168, 170–172)
HG106	Potent inhibition of SLC7A11, leading to reduced cystine uptake and GSH biosynthesis, with induction of oxidative and endoplasmic reticulum stress. The contribution of ferroptosis appears to be context dependent.	KRAS-mutant lung adenocarcinoma and hepatocellular carcinoma models and ovarian cancer patient-derived organoid and xenograft models	SLC7A11, cystine uptake, GSH, ROS, ER-stress markers, GPX4, and lipid peroxidation	Direct evidence in breast cancer and genetically defined BRCA1-mutant models is lacking. Pharmacokinetic and clinical safety data remain unavailable; pharmacodynamic assessment is confined to a limited set of KRAS-mutant lung and hepatocellular carcinoma preclinical models.	Define dose–response relationships, pharmacokinetics, target engagement, and cell-death mechanisms, and test HG106 in treatment-resistant BRCA1-mutant breast cancer models.	Extrapolated	(23, 24, 143, 174–176)
NRF2–SLC7A11 inhibition	Suppress NRF2-driven antioxidant transcription and SLC7A11-dependent ferroptosis defense, thereby increasing oxidative stress and lipid peroxidation. Small-molecule NRF2 inhibitors such as brusatol promote NRF2 ubiquitination and degradation, reducing NRF2 protein stability and transcriptional activity.	Paclitaxel-resistant breast cancer models; other breast cancer models; and bladder cancer models; breast cancer stem-like (mammosphere) models	NRF2, KEAP1, SLC7A11, GPX4, ROS, and lipid peroxidation	Pharmacological NRF2 inhibition remains challenging. Brusatol can enhance treatment responses, but its specificity and therapeutic window are limited. In breast cancer models, brusatol studies have primarily addressed NRF2/HO-1 signaling, apoptosis, or therapeutic resistance rather than NRF2–SLC7A11-dependent ferroptosis. Direct validation in genetically defined BRCA1-mutant breast cancer models is lacking.	Compare genetic NRF2/SLC7A11 suppression with selective pharmacological approaches and determine whether NRF2, KEAP1, SLC7A11, or GPX4 status predicts response in treatment-resistant BRCA1-mutant models.	Subtype-inferred	(180–189)
ATF4-pathway inhibition	Inhibit the PERK/GCN2–eIF2α–ATF4 stress-response pathway or ATF4-dependent transcription, thereby reducing SLC7A11 expression and related antioxidant programs.	Radioresistant and paclitaxel-treated TNBC models, with additional evidence from colorectal, pancreatic, renal, and hepatocellular carcinoma models	ATF4, p-eIF2α, SLC7A11, GSH, ROS, and lipid peroxidation	Broad ISR or PERK inhibition may cause pathway-related toxicity and impair normal stress adaptation. The role of ATF4 is context dependent and may be protective in some tissues.	Develop selective ATF4-pathway inhibitors and determine whether their combination with platinum agents or PARP inhibitors improves responses in treatment-resistant BRCA1-mutant breast cancer models.	Subtype-inferred	(55, 81, 176–179)
G6PD/PPP inhibition	Inhibit G6PD-dependent pentose phosphate pathway activity, thereby reducing NADPH regeneration and weakening GSH-dependent redox buffering, particularly in SLC7A11-high cells.	SLC7A11-high pan-cancer models; breast cancer and TNBC cell/xenograft models; broader G6PD/PPP cancer contexts	G6PD, PPP activity, NADPH/NADP+, ROS, GSH, SLC7A11, and lipid peroxidation	The prototype G6PD inhibitor 6-aminonicotinamide (6-AN) has limited translational suitability. Its activity has not been directly validated in BRCA1-mutant or platinum/PARP inhibitor-resistant breast cancer models.	Determine whether SLC7A11-high BRCA1-deficient tumors are selectively dependent on NADPH regeneration and evaluate combinations with SLC7A11 inhibitors, platinum agents, or PARP inhibitors.	Subtype-inferred	(17, 18, 20, 94, 95, 190–194)
Glucose/GLUT1 targeting	Restrict glucose uptake or utilization and thereby reduce glucose-dependent NADPH regeneration. In SLC7A11-high cells, this may induce disulfide stress, F-actin collapse, and disulfidptosis.	SLC7A11-high pan-cancer models; breast cancer and GLUT1-targeting models; pan-cancer disulfidptosis models	SLC7A11, GLUT1, NADPH/NADP+, GSH, disulfide stress, F-actin integrity, and INF2	Direct evidence for disulfidptosis in BRCA1-deficient breast cancer is lacking. Systemic inhibition of glucose metabolism may impair normal glucose-dependent tissues and narrow the therapeutic window.	Determine whether SLC7A11-high, BRCA1-deficient breast tumors show selective sensitivity to glucose/GLUT1 restriction and distinguish disulfidptosis from canonical ferroptosis.	Subtype-inferred	(17–19, 24, 42, 95, 99, 103–107, 195–209)

SLC7A11, solute carrier family 7 member 11 (xCT); GPX4, glutathione peroxidase 4; GSH, glutathione; ROS, reactive oxygen species; VDAC3, voltage-dependent anion channel 3; PPP, pentose phosphate pathway; G6PD, glucose-6-phosphate dehydrogenase; GLUT1, glucose transporter 1; NADPH/NADP+, reduced/oxidized nicotinamide adenine dinucleotide phosphate; NRF2, nuclear factor erythroid 2-related factor 2; KEAP1, Kelch-like ECH-associated protein 1; ATF4, activating transcription factor 4; eIF2α, eukaryotic initiation factor 2α; PERK, protein kinase R-like endoplasmic reticulum kinase; GCN2, general control nonderepressible 2; ISR, integrated stress response; ER, estrogen receptor; INF2, inverted formin 2; RAF/MEK/ERK, rapidly accelerated fibrosarcoma/mitogen-activated protein kinase kinase/extracellular signal-regulated kinase; HO-1, heme oxygenase-1; F-actin, filamentous actin; 6-AN, 6-aminonicotinamide; TNBC, triple-negative breast cancer; PARP, poly(ADP-ribose) polymerase. Each row is assigned one of the three evidence statuses (“Direct,” “Subtype-inferred,” or “Extrapolated”) defined in the **Table 1** footnote; the Supporting model/context column lists the models on which each assignment is based. None of the rows currently meets the criteria for “Direct” status. Where reported in the primary studies, the BRCA1 background of the supporting models is indicated; otherwise, it is identified as uncharacterized.

**Figure 4:**
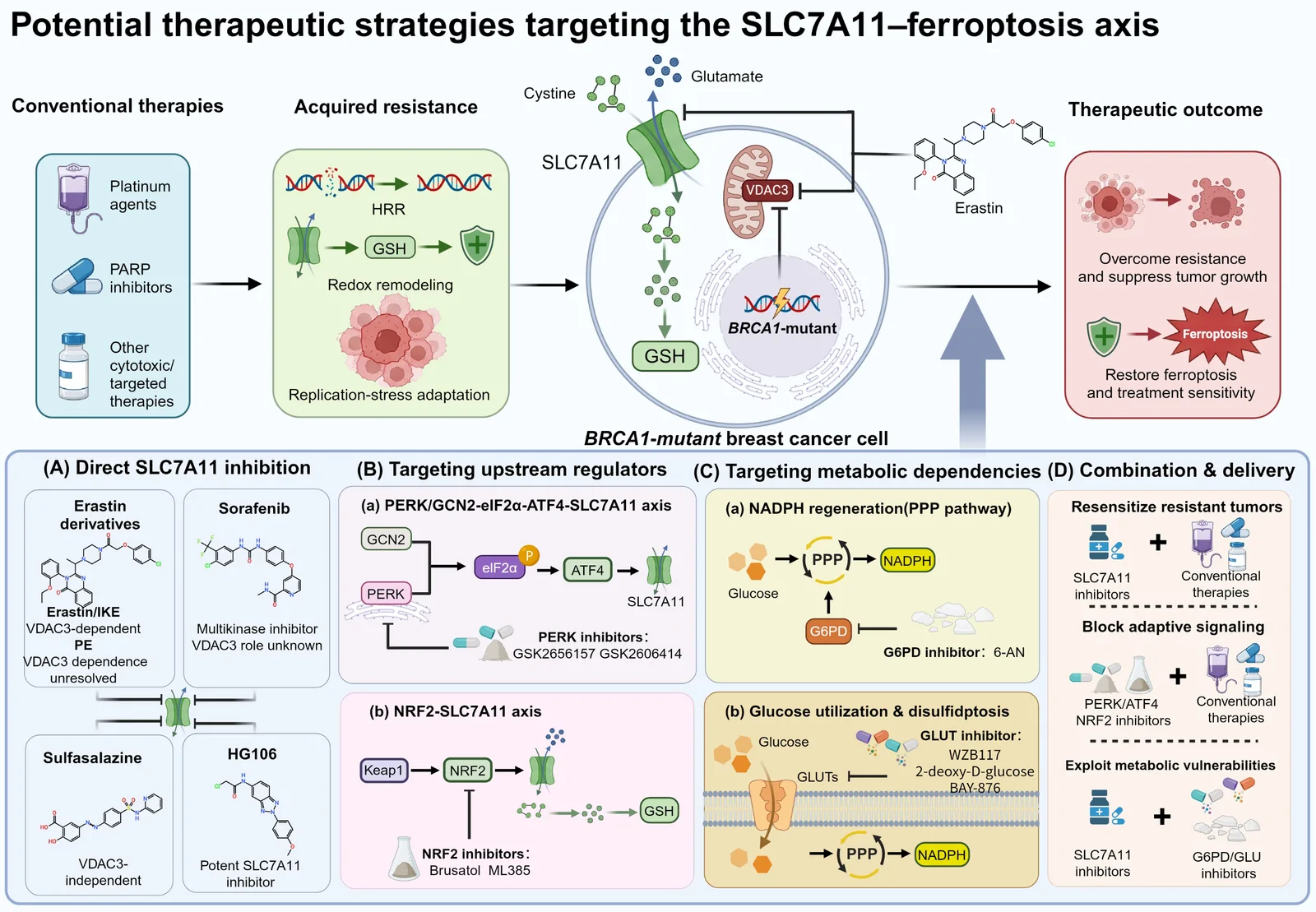
Proposed therapeutic strategies targeting the SLC7A11–ferroptosis axis after acquired treatment resistance in BRCA1-mutant breast cancer. **(A)** Direct SLC7A11 inhibition, **(B)** targeting upstream regulators, **(C)** targeting metabolic dependencies, and **(D)** combination and delivery strategies are depicted in separate panels. Conventional therapies may select for acquired resistance through HRR restoration, replication-stress adaptation, and redox remodeling. Candidate approaches include direct SLC7A11 inhibition; blockade of PERK/GCN2–eIF2α–ATF4 or NRF2 signaling; and targeting G6PD/PPP-dependent NADPH regeneration or GLUT-mediated glucose utilization to induce disulfidptosis. Combination and delivery strategies may enhance ferroptotic activity and potentially resensitize tumor cells. VDAC3 may influence responses to erastin-based inducers, whereas sulfasalazine is VDAC3-independent; VDAC3 involvement in sorafenib response and HG106 activity in breast cancer remains unresolved. Most strategies are preclinical and have not been directly validated in genetically defined, treatment-resistant BRCA1-mutant models. Created with BioRender.com.

### 3.1 Preclinical barriers to targeting SLC7A11 *in vivo*

Several preclinical barriers may limit the therapeutic efficacy of SLC7A11 inhibition *in vivo*. First, tumor cells may compensate for SLC7A11 inhibition by engaging SLC7A11-independent cysteine supply routes, including the transsulfuration pathway and macropinocytosis-dependent scavenging of extracellular proteins (139), and by upregulating parallel ferroptosis-defense systems, most notably FSP1–CoQ10 and DHODH (139, 140). These adaptations may preserve GPX4-dependent lipid-peroxide detoxification and thereby reduce the efficacy of SLC7A11 inhibition. Second, the pharmacological properties of currently available SLC7A11 inhibitors remain limiting. Erastin and its derivatives may have poor solubility, metabolic instability, or dependence on VDAC3, whereas sulfasalazine has relatively low potency, limited bioavailability, and uncertain tumor penetration. Third, tumor-microenvironmental interactions among cancer cells, cancer-associated fibroblasts, immune cells, and vascular compartments can reshape the local availability of cystine, cysteine, and glutathione and thereby influence responses to SLC7A11 inhibition (116, 141, 142). CAF-derived cysteine in particular can sustain tumor-cell glutathione synthesis and contribute to ferroptosis resistance (116), whereas fibrosis-associated vascular abnormalities may create hypoxic and nutrient-limited niches in which these metabolic interactions occur (141). These stromal and metabolic adaptations may reduce target dependence or protect tumor cells from ferroptosis after treatment. Fourth, systemic inhibition of cystine utilization or glucose metabolism may affect normal tissues and narrow the therapeutic window. Therefore, successful *in vivo* targeting will require pharmacodynamic confirmation of SLC7A11 inhibition, assessment of compensatory redox pathways, evaluation of tumor and stromal compartments, and rational combination or tumor-selective delivery strategies. These requirements should be addressed before SLC7A11-directed approaches are advanced toward clinical testing in BRCA1-mutant breast cancer.

### 3.2 Direct SLC7A11 inhibition and the VDAC3 constraint

Direct SLC7A11-targeting agents include erastin and its derivatives, sulfasalazine, sorafenib, and HG106 (26, 48, 143, 144). Their potential utility after treatment resistance depends on drug-specific mechanisms, VDAC3 status, pharmacological properties, and the resistance context. Erastin-like agents are limited by the reduced VDAC3 expression associated with BRCA1 deficiency, whereas the VDAC3 dependence of sorafenib remains unresolved and HG106 has not been evaluated in breast cancer models (24, 48, 144). Collectively, erastin derivatives, sulfasalazine, sorafenib, and HG106 represent mechanistically distinct SLC7A11-targeted candidates for investigation after conventional-treatment resistance, with combination and delivery strategies being explored to improve their therapeutic utility. However, whether any of these approaches can reverse platinum or PARP inhibitor resistance in BRCA1-mutant breast cancer, and whether any observed benefit depends on SLC7A11 inhibition rather than parallel ferroptosis-related mechanisms, remains unresolved.

#### 3.2.1 Erastin-based ferroptosis inducers: VDAC3 dependence and therapeutic limitations

Erastin, a canonical type I ferroptosis inducer, inhibits SLC7A11-dependent cystine uptake, depletes intracellular GSH, and promotes lipid peroxidation (145–147). However, a previous study showed that BRCA1 deficiency downregulates VDAC3, a mediator required for erastin-induced mitochondrial lipid peroxidation, thereby reducing erastin sensitivity in BRCA1-deficient tumor models (24). The most direct mechanistic evidence for the VDAC3–erastin chain was obtained in the HT1080 fibrosarcoma model; although Lei et al. also evaluated ovarian, triple-negative breast cancer, and BRCA1-mutant breast cancer xenograft models, the complete chain has not been independently reproduced in additional genotype-defined BRCA1-mutant breast cancer models (24). We therefore describe VDAC3 as a proposed, context-specific determinant of erastin response rather than an established universal mechanism of resistance. Poor water solubility and metabolic instability further limit the bioavailability and therapeutic development of erastin (148–150). Although piperazine erastin (PE) and imidazole ketone erastin (IKE) exhibit improved solubility and pharmacological properties (98, 151–153), IKE activity may also depend on VDAC3 in the reported models, and whether PE can overcome VDAC3-associated resistance remains unknown (24). Therefore, erastin and its derivatives may have limited efficacy as monotherapy approaches in BRCA1-mutant breast cancer.

Combination and delivery strategies may broaden the therapeutic utility of erastin-based ferroptosis induction. In ER-positive breast cancer models, tamoxifen enhances erastin- or IKE-induced ferroptosis by inhibiting ERα-mediated transcriptional activation of SLC7A11 and SLC3A2 (36). In TNBC models, MnFe_2_O_4_-erastin-perfluoropentane nanoparticles enable magnetically guided, near-infrared-responsive erastin release, promoting ferroptosis and immunogenic cell death (154). In HER2-positive breast cancer models, Fe-MOF@Erastin@Herceptin enables trastuzumab-directed erastin delivery and suppresses tumor growth and lung metastasis (155). However, these approaches have not been shown to bypass VDAC3-associated resistance. Their relevance to BRCA1-mutant breast cancer therefore requires validation in genotype-defined models, particularly in combination with strategies that restore or circumvent the VDAC3-dependent response.

#### 3.2.2 Sorafenib: SLC7A11-associated ferroptosis and therapeutic limitations

Sorafenib is an FDA-approved multikinase inhibitor targeting RAF kinases, VEGFRs, PDGFR, and other kinases (156–159); however, it is not approved for breast cancer. In addition to inhibiting the RAF/MEK/ERK pathway and exerting anti-angiogenic effects (143), sorafenib can induce ferroptosis by inhibiting system xc^−^/SLC7A11-mediated cystine uptake, depleting GSH, and promoting lipid peroxidation (160–162). This response is accompanied by mitochondrial ROS and lipid-peroxidation accumulation and has been reported in several cancer types, particularly hepatocellular carcinoma (163–165). However, the contribution of RAF/MEK/ERK signaling and VDAC3 to sorafenib-induced ferroptosis has not been directly established. Whether sorafenib can overcome the VDAC3-associated limitation observed with erastin in BRCA1-deficient tumors therefore remains unknown. In non-BRCA1-stratified TNBC models, sorafenib monotherapy showed limited antitumor activity, with tumor-inhibition rates below 40%. Its low oral bioavailability (approximately 8.43%) and potential hepatotoxicity may further restrict its development as a single-agent therapy (166). Thus, its therapeutic value in BRCA1-mutant breast cancer requires validation in genotype-defined models, including assessment of VDAC3 status, ferroptosis engagement, and treatment response.

These limitations have motivated preclinical evaluation of localized-delivery and combination strategies to improve sorafenib exposure and antitumor efficacy. In TNBC models, an injectable magnetothermal-responsive IMSFs hydrogel enabled localized sorafenib release and enhanced ferroptosis through the AMPK–Beclin1–SLC7A11 axis and Fe²^+^-mediated Fenton reactions (166). In ER-positive breast cancer models, sorafenib combined with tamoxifen showed synergistic antitumor activity, providing proof-of-concept for endocrine–ferroptosis combinations (36). The phase II LINUX platform trial (NCT05594095) has reported primary results for SNF subtype-guided therapy in patients with HR-positive/HER2-negative advanced breast cancer (Fan et al., *Cancer Cell* 2026;44:355–365.e3). Sorafenib, although listed in the registry, was not included in the published regimens, and no BRCA1-stratified results were reported. Thus, the published findings do not establish the efficacy of sorafenib or SLC7A11-directed therapy in BRCA1-mutant breast cancer. ClinicalTrials.gov lists the trial as recruiting, with an estimated enrollment of 620 participants; however, because the record was last updated in October 2024, this status may not reflect the trial’s current recruitment state. No results for the registry-listed sorafenib-containing SNF4 4D arm are posted.

#### 3.2.3 Sulfasalazine: VDAC3-independent SLC7A11 inhibition and combination strategies

Sulfasalazine is an FDA-approved anti-inflammatory drug that inhibits SLC7A11 activity independently of VDAC3 (24, 167). Despite its relatively low potency, limited bioavailability, and insufficient tumor penetration, sulfasalazine has shown preclinical activity in TNBC models by targeting SLC7A11-associated redox adaptation (32, 153, 168–171). In TNBC models, sulfasalazine depletes intracellular GSH and increases ROS accumulation, thereby weakening SLC7A11-associated ferroptosis defense and suppressing tumor growth (32). Complementary work by Long et al. showed that sulfasalazine downregulates SLC7A11, MYC, CDK1, and CD44 and activates mTOR-associated autophagy, resulting in reduced TNBC cell proliferation and tumor progression (171). Thus, sulfasalazine may impair both redox defense and oncogenic signaling in TNBC.

Given its limited potency as a monotherapy, sulfasalazine has been evaluated in combination regimens aimed at enhancing treatment response and testing whether SLC7A11-associated redox adaptation contributes to therapy-associated resistance. Metformin synergized with sulfasalazine to induce ferroptosis in ER-positive (T47D and MCF7) and TNBC (MDA-MB-231) breast cancer cell lines (172). In TNBC models, rapamycin combined with sulfasalazine enhanced autophagy and suppressed CD44 and SLC7A11, potentially targeting the chemoresistance-associated CD44v9–SLC7A11 axis (171). Sulfasalazine also enhanced the effects of IGF-1R inhibition in ER-positive models (173) and reduced the carboplatin IC_50_ in 13 of 14 TNBC samples, supporting SLC7A11 inhibition as a strategy to increase platinum sensitivity (32). These findings support sulfasalazine-based combinations as candidate approaches for treatment-refractory breast cancer, although their efficacy in BRCA1-mutant or PARP inhibitor-resistant models remains to be established.

#### 3.2.4 HG106: a potent SLC7A11 inhibitor with preclinical activity

Current evidence for HG106 is limited to non-breast cancer models. In KRAS-mutant lung adenocarcinoma models, HG106 acts as a potent SLC7A11 inhibitor that suppresses cystine uptake, increases ROS accumulation, and induces oxidative- and endoplasmic-reticulum-stress-mediated apoptosis. At approximately 10 μM, HG106 produces effects comparable to those of sulfasalazine at 1 mM, indicating roughly 100-fold greater *in vitro* potency (174). HG106 showed preclinical antitumor activity in lung and ovarian cancer models (174, 175). In ovarian cancer models, HG106 enhanced cisplatin activity and increased platinum sensitivity through inhibition of the SLC7A11-associated resistance pathway (175). Additionally, HG106 combined with the KRAS inhibitor NSC48160 suppressed the NRF2–SLC7A11–GPX4 axis, enhanced ferroptosis, and inhibited tumor growth in hepatocellular carcinoma models (176). Together, these findings support HG106 as a candidate strategy for targeting SLC7A11-dependent redox adaptation and treatment resistance. However, its activity in BRCA1-mutant breast cancer, particularly in VDAC3-low or PARP inhibitor-resistant models, remains untested and warrants further investigation (24).

### 3.3 Targeting upstream and regulatory nodes controlling SLC7A11 in treatment-resistant disease

#### 3.3.1 PERK/GCN2–eIF2α–ATF4–SLC7A11 signaling in therapeutic adaptation

In TNBC cell lines (MDA-MB-231 and BT-549) and breast cancer specimens, paclitaxel activates PERK/GCN2–eIF2α–ATF4 signaling, transcriptionally upregulating SLC7A11 and promoting cell survival during treatment (81). Inhibition of eIF2α signaling or ATF4 reduces SLC7A11 expression and sensitizes cells to paclitaxel, establishing an SLC7A11-linked chemotherapy-adaptation mechanism in breast cancer (81). However, this functional relationship has not been directly validated in genotype-defined BRCA1-mutant breast cancer models. Given the frequent TNBC phenotype of BRCA1-mutant breast cancer, this pathway warrants evaluation in BRCA1-mutant disease. Its therapeutic tractability is further supported by other cancer models. The PERK inhibitor GSK2656157 downregulates SLC7A11 and sensitizes colorectal cancer cells to ferroptosis (177), whereas GSK2606414 or MCU inhibition restores gemcitabine/nab-paclitaxel sensitivity in chemoresistant pancreatic cancer through the MCU–PERK–eIF2α–ATF4/NRF2 pathway (178). In renal cell carcinoma, salinomycin-mediated PDIA4 targeting suppresses ATF4–SLC7A11-dependent ferroptosis resistance (179). Although untested in BRCA1-mutant, platinum-resistant, or PARP inhibitor-resistant breast cancer models, these findings support targeting stress-response–SLC7A11 regulatory pathways as a candidate strategy for restoring treatment sensitivity.

#### 3.3.2 NRF2–SLC7A11 signaling in acquired ferroptosis resistance

The NRF2–SLC7A11–GSH–GPX4 axis may contribute to acquired ferroptosis resistance during chemotherapy adaptation. In paclitaxel-resistant breast cancer models, miR-141-3p suppresses KEAP1 and activates NRF2-dependent SLC7A11–GSH–GPX4 signaling, thereby limiting ferroptosis and promoting resistance. Pharmacological NRF2 inhibition with ML385 reverses these effects and restores ferroptotic sensitivity (180). This provides breast cancer-specific evidence that NRF2-directed interventions may help restore treatment sensitivity, although BRCA1-mutant models were not examined.

Brusatol is a widely investigated small-molecule NRF2 inhibitor and promotes NRF2 ubiquitination and degradation, thereby reducing NRF2 stability and transcriptional activity (181–183). In bladder cancer models, brusatol downregulates NRF2 and SLC7A11, depletes intracellular GSH, and induces ferroptosis (184). In breast cancer, however, brusatol has mainly been studied in relation to NRF2/HO-1 signaling, apoptosis, or therapeutic resistance in luminal, HER2-positive, and TNBC models (185–188), without directly establishing NRF2–SLC7A11-mediated ferroptosis (189). Thus, although NRF2 inhibition provides a mechanistically plausible approach to weaken SLC7A11-associated redox defense, the ferroptosis-specific activity of brusatol in breast cancer remains unresolved.

Collectively, these findings support evaluation of NRF2–SLC7A11-directed strategies as candidate approaches for treatment-refractory BRCA1-mutant breast cancer. Whether NRF2 inhibition can restore platinum or PARP inhibitor sensitivity through SLC7A11-dependent ferroptosis in genotype-defined resistant models remains to be determined.

### 3.4 Targeting metabolic dependencies created by SLC7A11 activity

Sustained SLC7A11 activity supports GSH–GPX4-mediated antioxidant defense but also increases the demand for NADPH regeneration. This metabolic dependency provides a rationale for targeting the pentose phosphate pathway, glucose utilization, or disulfide-stress responses as potential strategies for treatment-refractory BRCA1-mutant breast cancer.

#### 3.4.1 Targeting NADPH regeneration: G6PD inhibition as a candidate metabolic vulnerability

Glucose-6-phosphate dehydrogenase (G6PD), the rate-limiting enzyme of the pentose phosphate pathway, generates NADPH required for cellular redox maintenance (95, 190). In breast cancer, G6PD is frequently upregulated and has been associated with tumor progression, treatment resistance, and poor clinical outcomes (191). The classical G6PD inhibitor 6-aminonicotinamide (6-AN) reduces NADPH regeneration and disrupts redox homeostasis (192). In MCF-7 breast cancer cells, 6-AN induces ROS-associated autophagic cell death, whereas its *in vivo* antitumor activity has been demonstrated in MDA-MB-231 xenografts (193). In TNBC models, 6-AN inhibits tumor-cell proliferation and attenuates G6PD-dependent CCL2/TGF-β1 signaling and tumor-promoting macrophage interactions (194); its antiproliferative activity has also been observed in MDA-MB-231 cells (193).

These findings provide metabolic proof-of-concept, although 6-AN has not been tested in BRCA1-mutant or treatment-resistant breast cancer models. BRCA1 deficiency is associated with replication stress (20), altered mitochondrial metabolism (17, 18), and redox imbalance (18, 20), providing a rationale for combined targeting of SLC7A11-mediated cystine utilization and G6PD-dependent NADPH regeneration in treatment-refractory disease. Whether this strategy can enhance sensitivity to platinum agents or PARP inhibitors remains to be determined.

#### 3.4.2 Expanding therapeutic dimensions: targeting glucose metabolism and disulfidptosis

Disulfidptosis is a recently characterized form of regulated cell death that preferentially affects cells with high SLC7A11 expression (106, 195). Under glucose-restricted conditions, NADPH depletion limits the reduction of intracellular cystine to cysteine, thereby causing intracellular disulfide stress and promoting disulfidptotic cell death (106, 195). Recent mechanistic studies indicate that the Rac1–WAVE regulatory complex–Arp2/3 axis is involved in the assembly of branched actin networks during disulfidptosis (106, 196). The SLC7A11–INF2 axis has likewise been implicated in glucose deprivation-induced disulfidptosis: INF2 was identified as a component of a pan-cancer disulfidptosis-related gene signature, while functional studies in SLC7A11-high ovarian cancer cells showed that INF2 silencing attenuated disulfidptosis and preserved ATP and F-actin integrity (197). Disulfide cross-linking of actin cytoskeleton proteins, including filamin A/B (FLNA/FLNB) and non-muscle myosin II (MYH9), promotes F-actin contraction and detachment from the plasma membrane (106). Disulfidptosis is mechanistically distinct from ferroptosis and is not rescued by ferroptosis inhibitors; instead, it may be prevented or reversed by disulfide-reducing agents such as β-mercaptoethanol (2-ME) or tris(2-carboxyethyl)phosphine (TCEP) (106, 195). Together, these findings link SLC7A11-dependent cystine uptake and NADPH consumption to the cytoskeletal execution of disulfidptosis (106, 198).

Because high SLC7A11 activity imposes a substantial NADPH demand, SLC7A11-high tumors may depend heavily on glucose-derived NADPH (95, 106, 107) and become vulnerable to glucose restriction (105–107) or pharmacological GLUT inhibition (95, 106, 107). In SLC7A11-high models, BAY-876 and KL-11743 induce NADPH depletion, disulfide stress, and disulfidptosis by restricting glucose-dependent pentose phosphate pathway activity (106). Other glucose- or GLUT-targeting agents, including WZB117, 2-deoxy-D-glucose, phloretin, and apigenin, have shown metabolic or treatment-sensitizing activity in breast cancer models, but their ability to induce disulfidptosis has not been directly established (103, 199–202). In breast cancer models, WZB117 has been reported to enhance doxorubicin responses in MCF-7 cells and radiotherapy responses in MCF-7 and MDA-MB-231 cells, whereas 2-deoxy-D-glucose has shown antitumor activity in MDA-MB-231 models (199, 201).

BRCA1 deficiency is associated with altered mitochondrial and energetic metabolism (17, 18) and may increase cellular vulnerability to oxidative stress (18). In parallel, the SLC7A11–GSH–GPX4 system and NADPH-regenerating pathways constitute central components of cellular redox defense and ferroptosis control (19, 42, 99). Together with evidence that BRCA1 loss alters ferroptosis responses (24), these findings suggest that BRCA1-deficient tumors may be vulnerable to glucose restriction or disulfidptosis-inducing strategies. This vulnerability may be accentuated by the abnormal angiogenic and hypoxic tumor-microenvironment features reported in BRCA1-associated breast cancer (203, 204), which may generate regions of limited oxygen (203) and nutrient availability that constrain NADPH regeneration (205, 206). Because GLUT1 is ubiquitously expressed in normal tissues and provides basal glucose uptake in most cells, systemic inhibition of glucose transport may cause off-target toxicity and thereby narrow the therapeutic window (197, 200, 201). However, direct evidence for this vulnerability in BRCA1-mutant breast cancer remains unavailable.

##### 3.4.2.1 Glucose starvation as a convergence point for ferroptosis and disulfidptosis

Glucose starvation may produce overlapping but mechanistically separable ferroptotic and disulfidptotic phenotypes (106, 198). Reduced glucose availability limits pentose phosphate pathway flux and NADPH regeneration. NADPH depletion may simultaneously promote disulfide stress and impair GSH/GPX4-dependent lipid-peroxide detoxification, thereby favoring disulfidptosis and ferroptosis, respectively (106). These processes may coexist or occur sequentially in SLC7A11-high breast cancer models under glucose limitation and the accompanying NADPH/GSH redox stress (207). However, cystine deprivation cuts both ways: by limiting GSH synthesis it promotes ferroptosis (42, 106), whereas it attenuates disulfidptosis by reducing the cystine substrate available for intracellular disulfide accumulation (106). This mirror-image relationship represents the converse of the SLC7A11 paradox, in which the same transporter suppresses ferroptosis (42, 106) yet sensitizes glucose-starved cells to disulfidptosis (106, 196); consistent with recent syntheses that frame this duality as an SLC7A11-mediated “metabolic paradox” and “double-edged sword” linking the two regulated cell death programs (208).

##### 3.4.2.2 Experimental discrimination between ferroptosis and disulfidptosis in BRCA1-mutant models

Experimental validation should combine measurements of lipid peroxidation, labile iron, NADP+/NADPH ratios, intracellular disulfide accumulation, and F-actin organization with live-cell imaging and pathway-selective rescue experiments. Ferroptosis-selective rescue by ferrostatin-1, liproxstatin-1, or iron chelation (19, 106) should be contrasted with rescue by the disulfide-reducing agents 2-ME or TCEP (106), since only the latter fully suppressed glucose starvation-induced death in SLC7A11-high cells (106). Non-reducing immunoblotting for disulfide-linked FLNA, FLNB, or MYH9 species—which exhibit characteristic mobility shifts and high-molecular-weight smears—can provide orthogonal evidence of disulfidptotic execution (106, 207). Whether these death modes coexist or contribute differently to therapeutic sensitivity in genotype-defined BRCA1-mutant breast cancer remains unknown.

## 4 Future perspectives

Emerging evidence links SLC7A11-associated redox adaptation to ferroptosis resistance and treatment response in breast cancer, but its therapeutic relevance in BRCA1-mutant disease remains insufficiently defined. Future studies should therefore prioritize genotype-defined and treatment-resistant models, while integrating tumor-cell, immune, and stromal compartments.

### 4.1 Subtype-specific and spatial dissection of SLC7A11 regulation

Future studies should establish molecularly stratified cohorts of BRCA1-mutant breast cancer to define SLC7A11 expression, prognostic value, and regulatory circuitry, with emphasis on basal/TNBC tumors and comparison with less frequent luminal or HER2-positive subtypes (7, 34, 210). In ER-positive luminal breast cancer, SP1 binds the SLC7A11 promoter and modulates its expression, with functional assays linking this regulatory relationship to ferroptosis sensitivity (35). Integrative chromatin-accessibility profiling and single-cell multi-omics may help determine whether SP1 and other lineage-associated programs regulate SLC7A11 across breast cancer subtypes (211). Spatial transcriptomics, potentially complemented by spatial metabolomics, could help map regional relationships among SLC7A11 expression, redox state, and ferroptosis susceptibility in BRCA1-mutant breast cancer (24, 210, 212, 213). These efforts may support biomarker-guided development of SLC7A11-targeted strategies for treatment-resistant BRCA1-mutant breast cancer.

### 4.2 Adaptive resistance to SLC7A11-directed therapy

Prolonged SLC7A11 inhibition may select for compensatory antioxidant adaptations, including cysteine acquisition through transsulfuration or macropinocytosis and activation of alternative ferroptosis-defense systems such as FSP1 and DHODH (139). Because the SLC7A11–GSH–GPX4 and FSP1/DHODH–CoQ systems provide parallel ferroptosis defenses (140), combined targeting of SLC7A11 with GPX4 or other redox-protective nodes may help delay or overcome resistance to SLC7A11-directed therapy. Experimental support for such combination strategies currently comes primarily from SLC7A11/GPX4 co-targeting in sorafenib-resistant hepatocellular carcinoma models (214). Additionally, building on the metabolic rationale outlined in Section 3.4.2, disulfidptosis-inducing strategies may be explored as alternative or sequential treatments after SLC7A11 inhibitor failure. Although combined ferroptosis- and disulfidptosis-inducing approaches have shown preliminary activity in breast cancer models (207), their ability to overcome acquired resistance in BRCA1-mutant breast cancer remains unknown. This question should be examined in paired parental and resistant models, including SLC7A11 inhibitor-, platinum-, and PARP inhibitor-resistant systems. BRCA1 reversion mutations may represent another clinically relevant resistance context, but their effects on VDAC3, GPX4, SLC7A11 dependence, and ferroptosis sensitivity have not been directly established. Longitudinal analyses of reversion-positive tumors could determine whether BRCA1 restoration changes the response to SLC7A11- or ferroptosis-directed therapies.

### 4.3 Immune–metabolic regulation of SLC7A11 in treatment-resistant BRCA1-mutant breast cancer

SLC7A11 may influence treatment response through metabolic interactions between tumor cells and antitumor immune cells. Tumor cells with high SLC7A11 activity can outcompete CD8^+^ T cells for cystine by increasing SLC7A11-mediated cystine uptake, thereby promoting cystine deprivation, CD36-associated lipid peroxidation, ferroptosis, and functional T-cell exhaustion (215). Under NADPH-limited conditions, SLC7A11-mediated cystine uptake in CD8^+^ T cells has also been linked to disulfidptosis and T-cell exhaustion (216). Conversely, IFN-γ–JAK–STAT1 signaling can suppress SLC7A11 transcription in tumor cells, suggesting a potential means of reshaping tumor–T-cell metabolic interactions (217). Whether these mechanisms affect treatment resistance or immunotherapy response in BRCA1-mutant breast cancer remains unknown. Future studies should use cell-type-specific conditional models to distinguish the effects of SLC7A11 loss in tumor and immune compartments and evaluate SLC7A11-directed strategies in combination with immune checkpoint blockade, particularly in treatment-resistant BRCA1-mutant TNBC.

### 4.4 CAF-associated SLC7A11 as a potential stromal vulnerability

SLC7A11 is expressed in CAFs, and CAF-associated SLC7A11 activity has been linked to redox maintenance and tumor-supportive functions in non-breast cancer models (141, 142). In pancreatic cancer, CAFs rely on SLC7A11 for cystine uptake and glutathione synthesis, while its inhibition impairs CAF proliferation, collagen remodeling, and tumor-supportive activity; CAF-associated SLC7A11 expression is also linked to poor prognosis (141). In prostate cancer, the ZEB1–SLC7A11 axis protects iron-loaded CAFs from ferroptosis, and its disruption increases CAF ferroptosis sensitivity and reduces immunosuppressive activity (142). These findings provide a rationale for investigating whether CAF-associated SLC7A11 contributes to the stromal support of treatment resistance in BRCA1-mutant breast cancer, particularly in the inflammatory CAF-rich TNBC microenvironment. However, direct evidence in breast cancer and BRCA1-mutant, treatment-resistant models is currently lacking. Future studies should combine BRCA1-mutant tumor–CAF co-culture systems with cell-type-specific genetic perturbation to distinguish tumor-cell and stromal effects. If this stromal dependency is confirmed, CAF-directed delivery or coordinated targeting of tumor-cell and CAF-associated SLC7A11 could be evaluated as adjunctive strategies after platinum or PARP inhibitor resistance.

### 4.5 Biomarker-guided translation and clinical development

Breast cancer-specific preclinical studies provide initial support for the therapeutic relevance of SLC7A11 in treatment-resistant disease. SLC7A11 is elevated in trastuzumab-resistant HER2-positive tumors, whereas curcumenol enhances paclitaxel sensitivity in TNBC models by suppressing the SLC7A11/NF-κB/TGF-β pathway (39, 218). Although these findings were not obtained in BRCA1-mutant models, they support further evaluation of SLC7A11 as a therapeutic and pharmacodynamic marker in this setting. Clinical translation will require tumor-selective inhibitors or targeted delivery, rational combinations with chemotherapy or targeted agents, and biomarker-guided patient selection. Dynamic liquid-biopsy panels incorporating SLC7A11, GPX4, FSP1, lipid-peroxidation markers, and treatment-related genomic alterations could help identify patients most likely to benefit, although their predictive value requires prospective validation in genotype-defined, treatment-resistant models with careful assessment of target engagement, immune effects, and toxicity. Ultimately, SLC7A11-targeted therapy is best developed as a biomarker-guided approach for selected treatment-resistant BRCA1-mutant tumors rather than as a broadly applicable treatment.

## 5 Discussion

This review summarizes emerging evidence linking the SLC7A11–ferroptosis axis to redox adaptation, ferroptosis resistance, and treatment response in BRCA1-mutant breast cancer. BRCA1 deficiency may alter replication-stress responses, mitochondrial metabolism, and redox regulation, while therapeutic pressure can activate stress-responsive pathways such as ATF4 and NRF2 to reinforce SLC7A11-associated cystine utilization, glutathione synthesis, and GPX4-mediated antioxidant defense. SLC7A11 may therefore connect BRCA1-associated genomic instability with metabolic adaptation and treatment response, although its contribution to treatment resistance is likely context dependent and remains insufficiently defined. Thus, SLC7A11 should currently be regarded as a candidate stress-adaptive node rather than an established driver of chemotherapy or PARP inhibitor resistance in BRCA1-mutant breast cancer. The proposed “defense-as-consumption” model further suggests that enhanced SLC7A11 activity may increase dependence on NADPH and glucose metabolism, creating potential vulnerabilities to G6PD inhibition, glucose restriction, and disulfidptosis-inducing strategies. Under glucose restriction, NADPH depletion may promote disulfide stress while weakening GSH/GPX4-dependent lipid-peroxide detoxification, potentially linking disulfidptosis and ferroptosis; however, these responses have not been directly established in BRCA1-mutant breast cancer.

These findings help explain why reduced erastin sensitivity need not indicate broad ferroptosis resistance in BRCA1-deficient tumors. BRCA1 promotes VDAC3 and GPX4 transcription; in the reported models, BRCA1 loss reduced both proteins, blunted erastin-induced lipid peroxidation, and increased sensitivity to GPX4 inhibitors (24). Sulfasalazine inhibits SLC7A11 independently of VDAC3 (24), providing a rationale to test SLC7A11 inhibition when erastin sensitivity is reduced, although this has not been established in BRCA1-mutant breast cancer. In a separate mechanism, BRCA1/BARD1-mediated K6-linked ubiquitination and degradation of GPX4 may increase GPX4 abundance after BRCA1 loss in some settings (25). Together, these findings suggest that BRCA1-associated GPX4 regulation and ferroptosis responses are context dependent (24, 25). These mechanistic distinctions support evaluating VDAC3-dependent erastin response, the VDAC3-independent action of sulfasalazine on SLC7A11, and GPX4 inhibition as distinct ferroptosis-directed strategies. Their relative contributions require validation in genotype-defined BRCA1-mutant breast cancer models.

Several limitations constrain interpretation of the current evidence. Most studies remain preclinical, and a substantial proportion of mechanistic and therapeutic evidence derives from non-breast cancer models, including the STC2–PRMT5–SLC7A11 axis (46), the IMP2–IPO4–SLC7A11 pathway (115), HG106 (174–176), disulfidptosis and GLUT1-linked disulfidptosis mechanisms (195, 197, 202, 209), and PERK inhibition (177). These studies provide valuable mechanistic hypotheses but may not recapitulate the metabolic, lineage-specific, and treatment-adapted states of BRCA1-mutant breast cancer. Moreover, the activity of GLUT inhibitors has been investigated across several cancer types, including breast cancer, but their ability to induce SLC7A11-dependent disulfidptosis remains insufficiently defined in this disease context (199–201). BRCA1-deficient tumors display replication stress, altered mitochondrial metabolism, and context-dependent ferroptosis responses, while VDAC3 and GPX4 regulation may influence sensitivity to distinct ferroptosis-inducing agents (24). Direct validation in BRCA1-mutant models with acquired platinum or PARP inhibitor resistance is therefore essential.

The durability of SLC7A11-directed treatment may also be limited by tumor heterogeneity and compensatory redox pathways, including FSP1-, DHODH-, transsulfuration-, and macropinocytosis-dependent mechanisms (139). Future studies should determine whether these pathways are best targeted sequentially or in combination with SLC7A11 inhibition. Clinical translation will require tumor-selective inhibitors or delivery systems, rational combinations with platinum agents, PARP inhibitors, or other ferroptosis regulators, and dynamic biomarker panels incorporating SLC7A11, GPX4, FSP1, lipid-peroxidation markers, and treatment-related genomic alterations. These strategies should be evaluated in genotype-defined, treatment-resistant models with careful assessment of target engagement, immune effects, pharmacokinetics, and toxicity.

Overall, the SLC7A11–ferroptosis axis represents a biologically plausible and potentially actionable therapeutic node in treatment-resistant BRCA1-mutant breast cancer. Its clinical value will depend on identifying tumors that retain SLC7A11-associated vulnerabilities, defining the pathways that mediate adaptive resistance, and developing biomarker-guided combination strategies. Prospective validation in patient-derived models and clinical cohorts will be required to determine whether targeting this axis can improve outcomes in patients with treatment-resistant BRCA1-deficient breast cancer and whether any benefit is attributable specifically to SLC7A11 inhibition.

## References

[1] Siegel RL Kratzer TB Giaquinto AN Sung H Jemal A . Cancer statistics, 2025. CA Cancer J Clin. (2025) 75:10–45. doi: 10.3322/caac.21871 39817679 PMC11745215

[2] Bray F Laversanne M Sung H Ferlay J Siegel RL Soerjomataram I . Global cancer statistics 2022: GLOBOCAN estimates of incidence and mortality worldwide for 36 cancers in 185 countries. CA Cancer J Clin. (2024) 74:229–63. doi: 10.3322/caac.21834 38572751

[3] Dorling L Carvalho S Allen J González-Neira A Luccarini C Wahlström C . Breast cancer risk genes - association analysis in more than 113,000 women. N Engl J Med. (2021) 384:428–39. doi: 10.1056/NEJMoa1913948 33471991 PMC7611105

[4] Kuchenbaecker KB Hopper JL Barnes DR Phillips K-A Mooij TM Roos-Blom M-J . Risks of breast, ovarian, and contralateral breast cancer for BRCA1 and BRCA2 mutation carriers. JAMA. (2017) 317:2402–16. doi: 10.1001/jama.2017.7112 28632866

[5] Roy R Chun J Powell SN . BRCA1 and BRCA2: different roles in a common pathway of genome protection. Nat Rev Cancer. (2011) 12:68–78. doi: 10.1038/nrc3181 22193408 PMC4972490

[6] Biancolella M Testa B Baghernajad Salehi L D’Apice MR Novelli G . Genetics and genomics of breast cancer: update and translational perspectives. Semin Cancer Biol. (2021) 72:27–35. doi: 10.1016/j.semcancer.2020.03.013 32259642

[7] Trusler O Goodwin J Laslett AL . BRCA1 and BRCA2 associated breast cancer and the roles of current modelling systems in drug discovery. Biochim Biophys Acta Rev Cancer. (2021) 1875:188459. doi: 10.1016/j.bbcan.2020.188459 33129865

[8] Hustedt N Durocher D . The control of DNA repair by the cell cycle. Nat Cell Biol. (2016) 19:1–9. doi: 10.1038/ncb3452 28008184

[9] Farmer H McCabe N Lord CJ Tutt ANJ Johnson DA Richardson TB . Targeting the DNA repair defect in BRCA mutant cells as a therapeutic strategy. Nature. (2005) 434:917–21. doi: 10.1038/nature03445 15829967

[10] Ghanem A Domchek SM . New therapeutic options for BRCA mutant patients. Annu Rev Med. (2025) 76:175–87. doi: 10.1146/annurev-med-082523-083843 39630850

[11] Pilié PG Tang C Mills GB Yap TA . State-of-the-art strategies for targeting the DNA damage response in cancer. Nat Rev Clin Oncol. (2019) 16:81–104. doi: 10.1038/s41571-018-0114-z 30356138 PMC8327299

[12] Dias MP Moser SC Ganesan S Jonkers J . Understanding and overcoming resistance to PARP inhibitors in cancer therapy. Nat Rev Clin Oncol. (2021) 18:773–91. doi: 10.1038/s41571-021-00532-x 34285417

[13] Li H Liu Z-Y Wu N Chen Y-C Cheng Q Wang J . PARP inhibitor resistance: the underlying mechanisms and clinical implications. Mol Cancer. (2020) 19:107. doi: 10.1186/s12943-020-01227-0 32563252 PMC7305609

[14] Francica P Rottenberg S . Mechanisms of PARP inhibitor resistance in cancer and insights into the DNA damage response. Genome Med. (2018) 10:101. doi: 10.1186/s13073-018-0612-8 30593284 PMC6309079

[15] Kubli SP Bassi C Roux C Wakeham A Göbl C Zhou W . AhR controls redox homeostasis and shapes the tumor microenvironment in BRCA1-associated breast cancer. Proc Natl Acad Sci USA. (2019) 116:3604–13. doi: 10.1073/pnas.1815126116 30733286 PMC6397541

[16] Srinivas US Tan BWQ Vellayappan BA Jeyasekharan AD . ROS and the DNA damage response in cancer. Redox Biol. (2019) 25:101084. doi: 10.1016/j.redox.2018.101084 30612957 PMC6859528

[17] Yeo SK Paul R Haas M Wang C Guan J-L . Improved efficacy of mitochondrial disrupting agents upon inhibition of autophagy in a mouse model of BRCA1-deficient breast cancer. Autophagy. (2018) 14:1214–25. doi: 10.1080/15548627.2018.1460010 29938573 PMC6103709

[18] Privat M Radosevic-Robin N Aubel C Cayre A Penault-Llorca F Marceau G . BRCA1 induces major energetic metabolism reprogramming in breast cancer cells. PLoS One. (2014) 9:e102438. doi: 10.1371/journal.pone.0102438 25010005 PMC4092140

[19] Stockwell BR Friedmann Angeli JP Bayir H Bush AI Conrad M Dixon SJ . Ferroptosis: a regulated cell death nexus linking metabolism, redox biology, and disease. Cell. (2017) 171:273–85. doi: 10.1016/j.cell.2017.09.021 28985560 PMC5685180

[20] Taglialatela A Alvarez S Leuzzi G Sannino V Ranjha L Huang J-W . Restoration of replication fork stability in BRCA1- and BRCA2-deficient cells by inactivation of SNF2-family fork remodelers. Mol Cell. (2017) 68:e8.414–30. doi: 10.1016/j.molcel.2017.09.036 29053959 PMC5720682

[21] Jiang X Stockwell BR Conrad M . Ferroptosis: mechanisms, biology and role in disease. Nat Rev Mol Cell Biol. (2021) 22:266–82. doi: 10.1038/s41580-020-00324-8 33495651 PMC8142022

[22] Stockwell BR Jiang X Gu W . Emerging mechanisms and disease relevance of ferroptosis. Trends Cell Biol. (2020) 30:478–90. doi: 10.1016/j.tcb.2020.02.009 32413317 PMC7230071

[23] Lei G Zhuang L Gan B . The roles of ferroptosis in cancer: tumor suppression, tumor microenvironment, and therapeutic interventions. Cancer Cell. (2024) 42:513–34. doi: 10.1016/j.ccell.2024.03.011 38593779

[24] Lei G Mao C Horbath AD Yan Y Cai S Yao J . BRCA1-mediated dual regulation of ferroptosis exposes a vulnerability to GPX4 and PARP co-inhibition in BRCA1-deficient cancers. Cancer Discov. (2024) 14:1476–95. doi: 10.1158/2159-8290.CD-23-1220 38552003 PMC11296921

[25] Xie X Chen C Wang C Guo Y Sun B Tian J . Targeting GPX4-mediated ferroptosis protection sensitizes BRCA1-deficient cancer cells to PARP inhibitors. Redox Biol. (2024) 76:103350. doi: 10.1016/j.redox.2024.103350 39265497 PMC11415882

[26] Koppula P Zhuang L Gan B . Cystine transporter SLC7A11/xCT in cancer: ferroptosis, nutrient dependency, and cancer therapy. Protein Cell. (2021) 12:599–620. doi: 10.1007/s13238-020-00789-5 33000412 PMC8310547

[27] Zhu W-W Liu Y Yu Z Wang H-Q . SLC7A11-mediated cell death mechanism in cancer: a comparative study of disulfidptosis and ferroptosis. Front Cell Dev Biol. (2025) 13:1559423. doi: 10.3389/fcell.2025.1559423 40535572 PMC12174375

[28] Li X Zou L . BRCAness, DNA gaps, and gain and loss of PARP inhibitor-induced synthetic lethality. J Clin Invest. (2024) 134:e181062. doi: 10.1172/JCI181062 39007266 PMC11245158

[29] Jiang L Kon N Li T Wang S-J Su T Hibshoosh H . Ferroptosis as a p53-mediated activity during tumour suppression. Nature. (2015) 520:57–62. doi: 10.1038/nature14344 25799988 PMC4455927

[30] Zhang Y Shi J Liu X Feng L Gong Z Koppula P . BAP1 links metabolic regulation of ferroptosis to tumour suppression. Nat Cell Biol. (2018) 20:1181–92. doi: 10.1038/s41556-018-0178-0 30202049 PMC6170713

[31] Lu H Samanta D Xiang L Zhang H Hu H Chen I . Chemotherapy triggers HIF-1-dependent glutathione synthesis and copper chelation that induces the breast cancer stem cell phenotype. Proc Natl Acad Sci USA. (2015) 112:E4600–4609. doi: 10.1073/pnas.1513433112 26229077 PMC4547233

[32] Timmerman LA Holton T Yuneva M Louie RJ Padró M Daemen A . Glutamine sensitivity analysis identifies the xCT antiporter as a common triple-negative breast tumor therapeutic target. Cancer Cell. (2013) 24:450–65. doi: 10.1016/j.ccr.2013.08.020 24094812 PMC3931310

[33] Liu X Ma Z Jing X Wang G Zhao L Zhao X . The deubiquitinase OTUD5 stabilizes SLC7A11 to promote progression and reduce paclitaxel sensitivity in triple-negative breast cancer. Cancer Lett. (2024) 604:217232. doi: 10.1016/j.canlet.2024.217232 39276913

[34] Nath P Alfarsi LH El-Ansari R Masisi BK Erkan B Fakroun A . The amino acid transporter SLC7A11 expression in breast cancer. Cancer Biol Ther. (2024) 25:2291855. doi: 10.1080/15384047.2023.2291855 38073087 PMC10761065

[35] Cui Y Li Y Xu Y Liu X Kang X Zhu J . SLC7A11 protects luminal A breast cancer cells against ferroptosis induced by CDK4/6 inhibitors. Redox Biol. (2024) 76:103304. doi: 10.1016/j.redox.2024.103304 39153252 PMC11378944

[36] Cao J Zhou T Wu T Lin R Huang J Shi D . Targeting estrogen-regulated system xc^−^ promotes ferroptosis and endocrine sensitivity of ER+ breast cancer. Cell Death Dis. (2025) 16:30. doi: 10.1038/s41419-025-07354-0 39833180 PMC11756422

[37] Zou Y Zheng S Xie X Ye F Hu X Tian Z . N6-methyladenosine regulated FGFR4 attenuates ferroptotic cell death in recalcitrant HER2-positive breast cancer. Nat Commun. (2022) 13:2672. doi: 10.1038/s41467-022-30217-7 35562334 PMC9106694

[38] Conti L Bolli E Di Lorenzo A Franceschi V Macchi F Riccardo F . Immunotargeting of the xCT cystine/glutamate antiporter potentiates the efficacy of HER2-targeted immunotherapies in breast cancer. Cancer Immunol Res. (2020) 8:1039–53. doi: 10.1158/2326-6066.CIR-20-0082 32532810

[39] Hua Y Duan N Sun C Yang F Tian M Sun Y . Targeting SLC7A11-mediated cysteine metabolism for the treatment of trastuzumab-resistant HER2-positive breast cancer. Elife. (2025) 14:RP103953. doi: 10.7554/eLife.103953 40464376 PMC12136593

[40] Luo Y Akatsuka S Motooka Y Kong Y Zheng H Mashimo T . BRCA1 haploinsufficiency impairs iron metabolism to promote chrysotile-induced mesothelioma via ferroptosis resistance. Cancer Sci. (2023) 114:1423–36. doi: 10.1111/cas.15705 36541514 PMC10067434

[41] Hong T Lei G Chen X Li H Zhang X Wu N . PARP inhibition promotes ferroptosis via repressing SLC7A11 and synergizes with ferroptosis inducers in BRCA-proficient ovarian cancer. Redox Biol. (2021) 42:101928. doi: 10.1016/j.redox.2021.101928 33722571 PMC8113041

[42] Koppula P Zhang Y Zhuang L Gan B . Amino acid transporter SLC7A11/xCT at the crossroads of regulating redox homeostasis and nutrient dependency of cancer. Cancer Commun (Lond). (2018) 38:12. doi: 10.1186/s40880-018-0288-x 29764521 PMC5993148

[43] Lien EC Lyssiotis CA Juvekar A Hu H Asara JM Cantley LC . Glutathione biosynthesis is a metabolic vulnerability in PI(3)K/Akt-driven breast cancer. Nat Cell Biol. (2016) 18:572–8. doi: 10.1038/ncb3341 27088857 PMC4848114

[44] Liu J Xia X Huang P . xCT: a critical molecule that links cancer metabolism to redox signaling. Mol Ther. (2020) 28:2358–66. doi: 10.1016/j.ymthe.2020.08.021 32931751 PMC7647670

[45] Liu X Zhang Y Zhuang L Olszewski K Gan B . NADPH debt drives redox bankruptcy: SLC7A11/xCT-mediated cystine uptake as a double-edged sword in cellular redox regulation. Genes Dis. (2021) 8:731–45. doi: 10.1016/j.gendis.2020.11.010 34522704 PMC8427322

[46] Jiang K Yin X Zhang Q Yin J Tang Q Xu M . STC2 activates PRMT5 to induce radioresistance through DNA damage repair and ferroptosis pathways in esophageal squamous cell carcinoma. Redox Biol. (2023) 60:102626. doi: 10.1016/j.redox.2023.102626 36764215 PMC9929488

[47] Cobler L Zhang H Suri P Park C Timmerman LA . xCT inhibition sensitizes tumors to γ-radiation via glutathione reduction. Oncotarget. (2018) 9:32280–97. doi: 10.18632/oncotarget.25794 30190786 PMC6122354

[48] He J Wang X Chen K Zhang M Wang J . The amino acid transporter SLC7A11-mediated crosstalk implicated in cancer therapy and the tumor microenvironment. Biochem Pharmacol. (2022) 205:115241. doi: 10.1016/j.bcp.2022.115241 36084707

[49] Qiu H Liu J Shao N Zhao J Chen C Jiang Y . SLC7A11 as a bridge between ferroptosis and disulfidptosis: a promising target for tumor treatment. Cell Commun Signal. (2025) 23:460. doi: 10.1186/s12964-025-02447-x 41146208 PMC12557889

[50] Liu Y Su Z Tavana O Gu W . Understanding the complexity of p53 in a new era of tumor suppression. Cancer Cell. (2024) 42:946–67. doi: 10.1016/j.ccell.2024.04.009 38729160 PMC11190820

[51] Fan K Zhou S Ren Y Xiong J Wang H Fu Y . Epigenetic regulation of ACSL4 via H2A monoubiquitylation connects lipid metabolism to BAP1-mediated ferroptosis. Cell Death Differ. (2025) 33(6):1136–51. doi: 10.1038/s41418-025-01624-2 41310183 PMC13246907

[52] Fiore A Zeitler L Russier M Groß A Hiller M-K Parker JL . Kynurenine importation by SLC7A11 propagates anti-ferroptotic signaling. Mol Cell. (2022) 82:920–932.e7. doi: 10.1016/j.molcel.2022.02.007 35245456 PMC7617904

[53] Chen D Tavana O Chu B Erber L Chen Y Baer R . NRF2 is a major target of ARF in p53-independent tumor suppression. Mol Cell. (2017) 68:224–232.e4. doi: 10.1016/j.molcel.2017.09.009 28985506 PMC5683418

[54] Fan Z Wirth A-K Chen D Wruck CJ Rauh M Buchfelder M . Nrf2-Keap1 pathway promotes cell proliferation and diminishes ferroptosis. Oncogenesis. (2017) 6:e371. doi: 10.1038/oncsis.2017.65 28805788 PMC5608917

[55] Bai X Ni J Beretov J Wasinger VC Wang S Zhu Y . Activation of the eIF2α/ATF4 axis drives triple-negative breast cancer radioresistance by promoting glutathione biosynthesis. Redox Biol. (2021) 43:101993. doi: 10.1016/j.redox.2021.101993 33946018 PMC8111851

[56] He F Zhang P Liu J Wang R Kaufman RJ Yaden BC . ATF4 suppresses hepatocarcinogenesis by inducing SLC7A11 (xCT) to block stress-related ferroptosis. J Hepatol. (2023) 79:362–77. doi: 10.1016/j.jhep.2023.03.016 36996941 PMC11332364

[57] Wang L Liu Y Du T Yang H Lei L Guo M . ATF3 promotes erastin-induced ferroptosis by suppressing system Xc. Cell Death Differ. (2020) 27:662–75. doi: 10.1038/s41418-019-0380-z 31273299 PMC7206049

[58] Fu D Wang C Yu L Yu R . Induction of ferroptosis by ATF3 elevation alleviates cisplatin resistance in gastric cancer by restraining Nrf2/Keap1/xCT signaling. Cell Mol Biol Lett. (2021) 26:26. doi: 10.1186/s11658-021-00271-y 34098867 PMC8186082

[59] Chen X Wang Z Li C Zhang Z Lu S Wang X . SIRT1 activated by AROS sensitizes glioma cells to ferroptosis via induction of NAD+ depletion-dependent activation of ATF3. Redox Biol. (2024) 69:103030. doi: 10.1016/j.redox.2024.103030 38181705 PMC10791567

[60] Su Z Kon N Yi J Zhao H Zhang W Tang Q . Specific regulation of BACH1 by the hotspot mutant p53R175H reveals a distinct gain-of-function mechanism. Nat Cancer. (2023) 4:564–81. doi: 10.1038/s43018-023-00532-z 36973430 PMC10320414

[61] Igarashi K Nishizawa H Saiki Y Matsumoto M . The transcription factor BACH1 at the crossroads of cancer biology: From epithelial-mesenchymal transition to ferroptosis. J Biol Chem. (2021) 297:101032. doi: 10.1016/j.jbc.2021.101032 34339740 PMC8387770

[62] Su Z Liu Y Wang L Gu W . Regulation of SLC7A11 as an unconventional checkpoint in tumorigenesis through ferroptosis. Genes Dis. (2025) 12:101254. doi: 10.1016/j.gendis.2024.101254 39569390 PMC11577153

[63] Xiao X Yang Y Yang Y Zhao G . Lactate dehydrogenase B delactylation promotes gastric cancer metastasis via enhancing glutathione-mediated resistance to ferroptosis. Cell Biol Toxicol. (2026) 42:46. doi: 10.1007/s10565-026-10165-4 41760941 PMC12999720

[64] Zang X He X-Y Xiao C-M Lin Q Wang M-Y Liu C-Y . Circular RNA-encoded oncogenic PIAS1 variant blocks immunogenic ferroptosis by modulating the balance between SUMOylation and phosphorylation of STAT1. Mol Cancer. (2024) 23:207. doi: 10.1186/s12943-024-02124-6 39334380 PMC11438063

[65] Deng Y Qiu Y Li X Gong T Guo J Liang H . PDK4-driven lactate accumulation facilitates LPCAT2 lactylation to exacerbate sepsis-induced acute lung injury. Cell Death Differ. (2026) 33:557–73. doi: 10.1038/s41418-025-01585-6 41057687 PMC13035903

[66] Ghezelayagh TS Pennington KP Norquist BM Khasnavis N Radke MR Kilgore MR . Characterizing TP53 mutations in ovarian carcinomas with and without concurrent BRCA1 or BRCA2 mutations. Gynecol Oncol. (2021) 160:786–92. doi: 10.1016/j.ygyno.2020.12.007 33375991 PMC8491988

[67] Reedy MB Hang T Gallion H Arnold S Smith SA . Antisense inhibition of BRCA1 expression and molecular analysis of hereditary tumors indicate that functional inactivation of the p53 DNA damage response pathway is required for BRCA-associated tumorigenesis. Gynecol Oncol. (2001) 81:441–6. doi: 10.1006/gyno.2001.6190 11371136

[68] Loboda AP Adonin LS Zvereva SD Guschin DY Korneenko TV Telegina AV . BRCA mutations-the Achilles heel of breast, ovarian and other epithelial cancers. Int J Mol Sci. (2023) 24:4982. doi: 10.3390/ijms24054982 36902416 PMC10003548

[69] Punziano C Trombetti S Cesaro E Grosso M Faraonio R . Antioxidant systems as modulators of ferroptosis: Focus on transcription factors. Antioxidants (Basel). (2024) 13:298. doi: 10.3390/antiox13030298 38539832 PMC10967371

[70] Umans RA Martin J Harrigan ME Patel DC Chaunsali L Roshandel A . Transcriptional regulation of amino acid transport in glioblastoma multiforme. Cancers (Basel). (2021) 13:6169. doi: 10.3390/cancers13246169 34944790 PMC8699180

[71] Xia Z Kon N Gu AP Tavana O Gu W . Deciphering the acetylation code of p53 in transcription regulation and tumor suppression. Oncogene. (2022) 41:3039–50. doi: 10.1038/s41388-022-02331-9 35487975 PMC9149126

[72] Ouchi T Lee SW Ouchi M Aaronson SA Horvath CM . Collaboration of signal transducer and activator of transcription 1 (STAT1) and BRCA1 in differential regulation of IFN-gamma target genes. Proc Natl Acad Sci USA. (2000) 97:5208–13. doi: 10.1073/pnas.080469697 10792030 PMC25807

[73] Welcsh PL Lee MK Gonzalez-Hernandez RM Black DJ Mahadevappa M Swisher EM . BRCA1 transcriptionally regulates genes involved in breast tumorigenesis. Proc Natl Acad Sci USA. (2002) 99:7560–5. doi: 10.1073/pnas.062181799 12032322 PMC124283

[74] Dodson M Castro-Portuguez R Zhang DD . NRF2 plays a critical role in mitigating lipid peroxidation and ferroptosis. Redox Biol. (2019) 23:101107. doi: 10.1016/j.redox.2019.101107 30692038 PMC6859567

[75] Kubben N Zhang W Wang L Voss TC Yang J Qu J . Repression of the antioxidant NRF2 pathway in premature aging. Cell. (2016) 165:1361–74. doi: 10.1016/j.cell.2016.05.017 27259148 PMC4893198

[76] Rojo de la Vega M Chapman E Zhang DD . NRF2 and the hallmarks of cancer. Cancer Cell. (2018) 34:21–43. doi: 10.1016/j.ccell.2018.03.022 29731393 PMC6039250

[77] Wang S-F Chen M-S Chou Y-C Ueng Y-F Yin P-H Yeh T-S . Mitochondrial dysfunction enhances cisplatin resistance in human gastric cancer cells via the ROS-activated GCN2-eIF2α-ATF4-xCT pathway. Oncotarget. (2016) 7:74132–51. doi: 10.18632/oncotarget.12356 27708226 PMC5342041

[78] Costa-Mattioli M Walter P . The integrated stress response: From mechanism to disease. Science. (2020) 368:eaat5314. doi: 10.1126/science.aat5314 32327570 PMC8997189

[79] Tameire F Verginadis II Leli NM Polte C Conn CS Ojha R . ATF4 couples MYC-dependent translational activity to bioenergetic demands during tumour progression. Nat Cell Biol. (2019) 21:889–99. doi: 10.1038/s41556-019-0347-9 31263264 PMC6608727

[80] Sasaki H Sato H Kuriyama-Matsumura K Sato K Maebara K Wang H . Electrophile response element-mediated induction of the cystine/glutamate exchange transporter gene expression. J Biol Chem. (2002) 277:44765–71. doi: 10.1074/jbc.M208704200 12235164

[81] Chen L He J Zhou J Xiao Z Ding N Duan Y . EIF2A promotes cell survival during paclitaxel treatment *in vitro* and *in vivo*. J Cell Mol Med. (2019) 23:6060–71. doi: 10.1111/jcmm.14469 31211507 PMC6714208

[82] Derismahafi Z Farhud D Razavirad A Muhammadnejad A Jahanbin B Farivar S . NRF2 expression level and estrogen function in BRCA1-mutated breast cancer. BMC Cancer. (2025) 25:1622. doi: 10.1186/s12885-025-14781-x 41120975 PMC12542346

[83] Gorrini C Baniasadi PS Harris IS Silvester J Inoue S Snow B . BRCA1 interacts with Nrf2 to regulate antioxidant signaling and cell survival. J Exp Med. (2013) 210:1529–44. doi: 10.1084/jem.20121337 23857982 PMC3727320

[84] Qiu Z Lin Y Chen S Cao W Yang J Li R . NUP62 promotes breast cancer progression and inhibits ferroptosis by stabilizing NRF2 in a KEAP1-dependent way. iScience. (2026) 29:115484. doi: 10.1016/j.isci.2026.115484 42016308 PMC13092479

[85] Wang Y Liang Y Luo D Ye F Jin Y Wang L . EDEM1 inhibits endoplasmic reticulum stress to induce doxorubicin resistance through accelerating ERAD and activating Keap1/Nrf2 antioxidant pathway in triple-negative breast cancer. Res (Wash D C). (2025) 8:797. doi: 10.34133/research.0797 40735463 PMC12305169

[86] van Diest PJ van der Groep P van der Wall E . EGFR expression predicts BRCA1 status in patients with breast cancer. Clin Cancer Res. (2006) 12:670. doi: 10.1158/1078-0432.CCR-05-2098 16428516

[87] Kumaraswamy E Wendt KL Augustine LA Stecklein SR Sibala EC Li D . BRCA1 regulation of epidermal growth factor receptor (EGFR) expression in human breast cancer cells involves microRNA-146a and is critical for its tumor suppressor function. Oncogene. (2015) 34:4333–46. doi: 10.1038/onc.2014.363 25417703 PMC4739738

[88] Mlinarić M Lucić I Milković L da Silva IV Tartaro Bujak I Musani V . AQP3-dependent PI3K/Akt modulation in breast cancer cells. Int J Mol Sci. (2023) 24:8133. doi: 10.3390/ijms24098133 37175840 PMC10179317

[89] Niu Z Jin R Zhang Y Li H . Signaling pathways and targeted therapies in lung squamous cell carcinoma: mechanisms and clinical trials. Signal Transduct Target Ther. (2022) 7:353. doi: 10.1038/s41392-022-01200-x 36198685 PMC9535022

[90] Wang S Liu L Li X Li Q Wang Y Feng X . cDTL contributes to breast cancer progression through regulating redox homeostasis via affecting the function of system xc. Biochem Biophys Res Commun. (2025) 744:151196. doi: 10.1016/j.bbrc.2024.151196 39706052

[91] Grushko TA Dignam JJ Das S Blackwood AM Perou CM Ridderstråle KK . MYC is amplified in BRCA1-associated breast cancers. Clin Cancer Res. (2004) 10:499–507. doi: 10.1158/1078-0432.ccr-0976-03 14760071

[92] Shi P Hu B Liu Y Li X Li W Huang C . Long non-coding RNA NORAD suppresses erastin-induced ferroptosis in breast cancer by upregulating SLC7A11 via targeting the FUS/NR3C1 axis: experimental studies. Int J Surg. (2025) 111:4295–313. doi: 10.1097/JS9.0000000000002531 40405780

[93] Tian R Li D Lan C Xu H Ma X Chen D . Targeting the PRSS3-PAR2-ERK1/2 axis inhibits malignancy and regulates chemosensitivity and resistance through ferroptosis in breast cancer. Free Radic Biol Med. (2025) 241:642–58. doi: 10.1016/j.freeradbiomed.2025.10.003 41046946

[94] Xu T Liu H Ling N Chen D Dai J Chen W . OTUD3-mediated stabilization of SLC7A11 drives sunitinib resistance by suppressing ferroptosis in clear cell renal cell carcinoma. Cancer Lett. (2025) 632:217942. doi: 10.1016/j.canlet.2025.217942 40716486

[95] Liu X Olszewski K Zhang Y Lim EW Shi J Zhang X . Cystine transporter regulation of pentose phosphate pathway dependency and disulfide stress exposes a targetable metabolic vulnerability in cancer. Nat Cell Biol. (2020) 22:476–86. doi: 10.1038/s41556-020-0496-x 32231310 PMC7194135

[96] Carneiro TJ Carvalho ALMB Vojtek M Carmo IF Marques MMPM Diniz C . Disclosing a metabolic signature of cisplatin resistance in MDA-MB-231 triple-negative breast cancer cells by NMR metabolomics. Cancer Cell Int. (2023) 23:310. doi: 10.1186/s12935-023-03124-0 38057765 PMC10699005

[97] Viswanathan VS Ryan MJ Dhruv HD Gill S Eichhoff OM Seashore-Ludlow B . Dependency of a therapy-resistant state of cancer cells on a lipid peroxidase pathway. Nature. (2017) 547:453–7. doi: 10.1038/nature23007 28678785 PMC5667900

[98] Yang WS SriRamaratnam R Welsch ME Shimada K Skouta R Viswanathan VS . Regulation of ferroptotic cancer cell death by GPX4. Cell. (2014) 156:317–31. doi: 10.1016/j.cell.2013.12.010 24439385 PMC4076414

[99] Friedmann Angeli JP Schneider M Proneth B Tyurina YY Tyurin VA Hammond VJ . Inactivation of the ferroptosis regulator Gpx4 triggers acute renal failure in mice. Nat Cell Biol. (2014) 16:1180–94. doi: 10.1038/ncb3064 25402683 PMC4894846

[100] Zhang Y Swanda RV Nie L Liu X Wang C Lee H . mTORC1 couples cyst(e)ine availability with GPX4 protein synthesis and ferroptosis regulation. Nat Commun. (2021) 12:1589. doi: 10.1038/s41467-021-21841-w 33707434 PMC7952727

[101] Glibetic N Weichhaus M . Metabolic regulation of ferroptosis in breast cancer. Int J Mol Sci. (2025) 26:9686. doi: 10.3390/ijms26199686 41096949 PMC12524448

[102] Szulc A Woźniak M . Targeting ferroptosis as the Achilles’ heel of breast cancer: Mechanisms and therapeutic opportunities from a comprehensive systematic review. Int J Mol Sci. (2025) 26:9902. doi: 10.3390/ijms26209902 41155194 PMC12564450

[103] Lee SY Hwang C Kim SE Karmakar M Jung SJ Jang H . Glucose deprivation-driven concurrent induction of apoptosis/disulfidptosis/ferroptosis by tumor targeting nanoassemblies in triple-negative breast cancer therapy. Adv Funct Mater. (2026) 36:e76803. doi: 10.1002/adfm.7680342587375

[104] Yan Y Teng H Hang Q Kondiparthi L Lei G Horbath A . SLC7A11 expression level dictates differential responses to oxidative stress in cancer cells. Nat Commun. (2023) 14:3673. doi: 10.1038/s41467-023-39401-9 37339981 PMC10281978

[105] Shin C-S Mishra P Watrous JD Carelli V D’Aurelio M Jain M . The glutamate/cystine xCT antiporter antagonizes glutamine metabolism and reduces nutrient flexibility. Nat Commun. (2017) 8:15074. doi: 10.1038/ncomms15074 28429737 PMC5413954

[106] Liu X Nie L Zhang Y Yan Y Wang C Colic M . Actin cytoskeleton vulnerability to disulfide stress mediates disulfidptosis. Nat Cell Biol. (2023) 25:404–14. doi: 10.1038/s41556-023-01091-2 36747082 PMC10027392

[107] Zheng T Liu Q Xing F Zeng C Wang W . Disulfidptosis: a new form of programmed cell death. J Exp Clin Cancer Res. (2023) 42:137. doi: 10.1186/s13046-023-02712-2 37259067 PMC10230712

[108] Kang HJ Kim HJ Rih J-K Mattson TL Kim KW Cho C-H . BRCA1 plays a role in the hypoxic response by regulating HIF-1α stability and by modulating vascular endothelial growth factor expression. J Biol Chem. (2006) 281:13047–56. doi: 10.1074/jbc.M513033200 16543242

[109] Vázquez-Arreguín K Maddox J Kang J Park D Cano RR Factor RE . BRCA1 through its E3 ligase activity regulates the transcription factor Oct1 and carbohydrate metabolism. Mol Cancer Res. (2018) 16:439–52. doi: 10.1158/1541-7786.MCR-17-0364 29330289 PMC5835178

[110] Zatreanu D Robinson HMR Alkhatib O Boursier M Finch H Geo L . Polθ inhibitors elicit BRCA-gene synthetic lethality and target PARP inhibitor resistance. Nat Commun. (2021) 12:3636. doi: 10.1038/s41467-021-23463-8 34140467 PMC8211653

[111] Belan O Sebald M Adamowicz M Anand R Vancevska A Neves J . POLQ seals post-replicative ssDNA gaps to maintain genome stability in BRCA-deficient cancer cells. Mol Cell. (2022) 82:4664–4680.e9. doi: 10.1016/j.molcel.2022.11.008 36455556

[112] Ceccaldi R Liu JC Amunugama R Hajdu I Primack B Petalcorin MIR . Homologous-recombination-deficient tumours are dependent on Polθ-mediated repair. Nature. (2015) 518:258–62. doi: 10.1038/nature14184 25642963 PMC4415602

[113] Chen X Li J Kang R Klionsky DJ Tang D . Ferroptosis: machinery and regulation. Autophagy. (2021) 17:2054–81. doi: 10.1080/15548627.2020.1810918 32804006 PMC8496712

[114] Kim Y-J Kim D Illuzzi JL Delaplane S Su D Bernier M . S-glutathionylation of cysteine 99 in the APE1 protein impairs abasic endonuclease activity. J Mol Biol. (2011) 414:313–26. doi: 10.1016/j.jmb.2011.10.023 22024594 PMC3373313

[115] Yan Y Huang Z Zhu Z Wang Y Cao X Yang C . IMP2 drives chemoresistance by repressing cisplatin-induced apoptosis and ferroptosis via activation of IPO4 and SLC7A11 under hypoxia in bladder cancer. Cancer Cell Int. (2024) 24:386. doi: 10.1186/s12935-024-03570-4 39578867 PMC11583454

[116] Zhu Y Fang S Fan B Xu K Xu L Wang L . Cancer-associated fibroblasts reprogram cysteine metabolism to increase tumor resistance to ferroptosis in pancreatic cancer. Theranostics. (2024) 14:1683–700. doi: 10.7150/thno.89805 38389839 PMC10879865

[117] Nee K Ma D Nguyen QH Pein M Pervolarakis N Insua-Rodríguez J . Preneoplastic stromal cells promote BRCA1-mediated breast tumorigenesis. Nat Genet. (2023) 55:595–606. doi: 10.1038/s41588-023-01298-x 36914836 PMC10655552

[118] Lee CM Hwang Y Jeong JW Kim M Lee J Bae SJ . BRCA1 mutation promotes sprouting angiogenesis in inflammatory cancer-associated fibroblast of triple-negative breast cancer. Cell Death Discov. (2024) 10:5. doi: 10.1038/s41420-023-01768-5 38182557 PMC10770063

[119] Ryan D Paul BT Koziol J ElShamy WM . The pro- and anti-tumor roles of mesenchymal stem cells toward BRCA1-IRIS-overexpressing TNBC cells. Breast Cancer Res. (2019) 21:53. doi: 10.1186/s13058-019-1131-2 31014367 PMC6480921

[120] Porcelli L Iacobazzi RM Di Fonte R Serratì S Intini A Solimando AG . CAFs and TGF-β signaling activation by mast cells contribute to resistance to gemcitabine/nabpaclitaxel in pancreatic cancer. Cancers (Basel). (2019) 11:330. doi: 10.3390/cancers11030330 30866547 PMC6468868

[121] Kanemaru A Yamamoto K Kawaguchi M Fukushima T Lin C-Y Johnson MD . Deregulated matriptase activity in oral squamous cell carcinoma promotes the infiltration of cancer-associated fibroblasts by paracrine activation of protease-activated receptor 2. Int J Cancer. (2017) 140:130–41. doi: 10.1002/ijc.30426 27615543

[122] Kang K Wu Y Han C Wang L Wang Z Zhao A . Homologous recombination deficiency in triple-negative breast cancer: Multi-scale transcriptomics reveals distinct tumor microenvironments and limitations in predicting immunotherapy response. Comput Biol Med. (2023) 158:106836. doi: 10.1016/j.compbiomed.2023.106836 37031511

[123] Bryzek D Gasiorek A Kowalczyk D Santocki M Ciaston I Dobosz E . Non-classical neutrophil extracellular traps induced by PAR2-signaling proteases. Cell Death Dis. (2025) 16:109. doi: 10.1038/s41419-025-07428-z 39971938 PMC11840154

[124] Sun Q Zhong Y Sun C . Single-cell RNA sequencing reveals immune microenvironment heterogeneity in BRCA1-mutated and sporadic triple-negative breast cancer. Transl Oncol. (2026) 69:102805. doi: 10.1016/j.tranon.2026.102805 42119173 PMC13194165

[125] Yang Z Su W Wei X Qu S Zhao D Zhou J . HIF-1α drives resistance to ferroptosis in solid tumors by promoting lactate production and activating SLC1A1. Cell Rep. (2023) 42:112945. doi: 10.1016/j.celrep.2023.112945 37542723

[126] van der Groep P Bouter A Menko FH van der Wall E van Diest PJ . High frequency of HIF-1α overexpression in BRCA1 related breast cancer. Breast Cancer Res Treat. (2008) 111:475–80. doi: 10.1007/s10549-007-9817-z 18030615

[127] Tsvetkov P Coy S Petrova B Dreishpoon M Verma A Abdusamad M . Copper induces cell death by targeting lipoylated TCA cycle proteins. Science. (2022) 375:1254–61. doi: 10.1126/science.abf0529 35298263 PMC9273333

[128] Tolbatov I Marrone A . Ferroptosis and cuproptosis in cancer and neurodegeneration: A comprehensive review of modulation by iron and copper chelators and related agents. Biomolecules. (2026) 16:348. doi: 10.3390/biom16030348 41897286 PMC13024704

[129] Deng X Deng Z Zhu B Hu R Ma J Li C . Glutathione reductase deficiency potentiates the immunogenicity of ferroptosis and cuproptosis via amplified reactive oxygen species accumulation and cGAS-STING pathway activation. J Hematol Oncol. (2026) 19:61. doi: 10.1186/s13045-026-01840-4 42681644 PMC13531920

[130] Ge EJ Bush AI Casini A Cobine PA Cross JR DeNicola GM . Connecting copper and cancer: from transition metal signalling to metalloplasia. Nat Rev Cancer. (2022) 22:102–13. doi: 10.1038/s41568-021-00417-2 34764459 PMC8810673

[131] Lutsenko S . Dynamic and cell-specific transport networks for intracellular copper ions. J Cell Sci. (2021) 134:jcs240523. doi: 10.1242/jcs.240523 34734631 PMC8627558

[132] Helman SL Zhou J Fuqua BK Lu Y Collins JF Chen H . The biology of mammalian multi-copper ferroxidases. Biometals. (2023) 36:263–81. doi: 10.1007/s10534-022-00370-z 35167013 PMC9376197

[133] Ji C Steimle BL Bailey DK Kosman DJ . The ferroxidase hephaestin but not amyloid precursor protein is required for ferroportin-supported iron efflux in primary hippocampal neurons. Cell Mol Neurobiol. (2018) 38:941–54. doi: 10.1007/s10571-017-0568-z 29177638 PMC5884744

[134] Chen Z Jiang R Chen M Zheng J Chen M Braidy N . Multi-copper ferroxidase deficiency leads to iron accumulation and oxidative damage in astrocytes and oligodendrocytes. Sci Rep. (2019) 9:9437. doi: 10.1038/s41598-019-46019-9 31263155 PMC6603037

[135] Anderson CP Shen M Eisenstein RS Leibold EA . Mammalian iron metabolism and its control by iron regulatory proteins. Biochim Biophys Acta. (2012) 1823:1468–83. doi: 10.1016/j.bbamcr.2012.05.010 22610083 PMC3675657

[136] Matuszczak M Kiljańczyk A Marciniak W Derkacz R Stempa K Baszuk P . Antioxidant properties of zinc and copper-blood zinc-to copper-ratio as a marker of cancer risk BRCA1 mutation carriers. Antioxidants (Basel). (2024) 13:841. doi: 10.3390/antiox13070841 39061909 PMC11273827

[137] Jain A Barge A Parris CN . Combination strategies with PARP inhibitors in BRCA-mutated triple-negative breast cancer: overcoming resistance mechanisms. Oncogene. (2025) 44:193–207. doi: 10.1038/s41388-024-03227-6 39572842 PMC11746151

[138] Cybulla E Wallace S Meroni A Jackson J Agashe S Tennakoon M . A RAD18-UBC13-PALB2-RNF168 axis mediates replication fork recovery in BRCA1-deficient cancer cells. Nucleic Acids Res. (2024) 52:8861–79. doi: 10.1093/nar/gkae563 38943334 PMC11347138

[139] Wang J Guo D Jiang S Wu W Gao X . Targeting ferroptosis in cancer: from mechanistic insights to therapeutic approaches. Mol BioMed. (2026) 7:20. doi: 10.1186/s43556-026-00416-5 41772328 PMC12953855

[140] Wang Y Yu G Chen X . Mechanism of ferroptosis resistance in cancer cells. Cancer Drug Resist. (2024) 7:47. doi: 10.20517/cdr.2024.127 39624080 PMC11609146

[141] Sharbeen G McCarroll JA Akerman A Kopecky C Youkhana J Kokkinos J . Cancer-associated fibroblasts in pancreatic ductal adenocarcinoma determine response to SLC7A11 inhibition. Cancer Res. (2021) 81:3461–79. doi: 10.1158/0008-5472.CAN-20-2496 33980655

[142] Zhang Y Zhao H Xu P Liu K Du G Chen X . The Zeb1/system Xc^−^ axis safeguards iron-loaded cancer-associated fibroblasts against ferroptosis and promotes immunosuppression in prostate cancer. Cancer Lett. (2026) 639:218243. doi: 10.1016/j.canlet.2026.218243 41485685

[143] Sharma A Srivastava Y Prasad S Ghosh S Kumar RS Bharti AC . SLC7A11 as a molecular nexus of prognosis, resistance, and therapeutic roadmap in cancer: A systematic review and meta-analysis. Int J Biol Macromol. (2026) 335:149257. doi: 10.1016/j.ijbiomac.2025.149257 41308763

[144] Lee J Roh J-L . SLC7A11 as a gateway of metabolic perturbation and ferroptosis vulnerability in cancer. Antioxidants (Basel). (2022) 11:2444. doi: 10.3390/antiox11122444 36552652 PMC9774303

[145] Lei G Zhuang L Gan B . Targeting ferroptosis as a vulnerability in cancer. Nat Rev Cancer. (2022) 22:381–96. doi: 10.1038/s41568-022-00459-0 35338310 PMC10243716

[146] Gan B . How erastin assassinates cells by ferroptosis revealed. Protein Cell. (2023) 14:84–6. doi: 10.1093/procel/pwac007 36929006 PMC10019563

[147] Yan R Xie E Li Y Li J Zhang Y Chi X . The structure of erastin-bound xCT-4F2hc complex reveals molecular mechanisms underlying erastin-induced ferroptosis. Cell Res. (2022) 32:687–90. doi: 10.1038/s41422-022-00642-w 35352032 PMC9253326

[148] Chen H Lyu F Gao X . Advances in ferroptosis for castration-resistant prostate cancer treatment: novel drug targets and combination therapy strategies. Prostate Cancer Prostatic Dis. (2026) 29:36–46. doi: 10.1038/s41391-024-00933-w 39733054 PMC12909126

[149] Bai L Shen L Zhang Q Hu Z Tang Y Fu M . Accelerating small molecule ferroptosis regulator development with advances in biological functions. Med Res Rev. (2026) 46(4):853–95. doi: 10.1002/med.70034 41731907

[150] Muluh TA Fu Q Ai X Wang C Chen W Zheng X . Targeting ferroptosis as an advance strategy in cancer therapy. Antioxid Redox Signal. (2024) 41:616–36. doi: 10.1089/ars.2024.0608 38959114

[151] Zhang Y Tan H Daniels JD Zandkarimi F Liu H Brown LM . Imidazole ketone erastin induces ferroptosis and slows tumor growth in a mouse lymphoma model. Cell Chem Biol. (2019) 26:e9.623–33. doi: 10.1016/j.chembiol.2019.01.008 30799221 PMC6525071

[152] Ferrada L Barahona MJ Vera M Stockwell BR Nualart F . Dehydroascorbic acid sensitizes cancer cells to system xc^−^ inhibition-induced ferroptosis by promoting lipid droplet peroxidation. Cell Death Dis. (2023) 14:637. doi: 10.1038/s41419-023-06153-9 37752118 PMC10522586

[153] Stockwell BR Jiang X . The chemistry and biology of ferroptosis. Cell Chem Biol. (2020) 27:365–75. doi: 10.1016/j.chembiol.2020.03.013 32294465 PMC7204503

[154] Qiu Y Wu H Lin Z Chen J Tian S Wang Z . Magneto-photo-acoustic nanotheranostics orchestrate ferroptosis-immune cross talk for spatiotemporally amplified triple-negative breast cancer therapy. Biomater Res. (2025) 29:258. doi: 10.34133/bmr.0258 41080653 PMC12509091

[155] Gao J Ye T Miao H Liu M Wen L Tian Y . Antibody-functionalized iron-based nanoplatform for ferroptosis-augmented targeted therapy of HER2-positive breast cancer. Bioact Mater. (2025) 52:702–18. doi: 10.1016/j.bioactmat.2025.06.034 40641578 PMC12241818

[156] Li M Su Y Zhang F Chen K Xu X Xu L . A dual-targeting reconstituted high density lipoprotein leveraging the synergy of sorafenib and antimiRNA21 for enhanced hepatocellular carcinoma therapy. Acta Biomater. (2018) 75:413–26. doi: 10.1016/j.actbio.2018.05.049 29859368

[157] Roskoski R Sadeghi-Nejad A . Role of RET protein-tyrosine kinase inhibitors in the treatment RET-driven thyroid and lung cancers. Pharmacol Res. (2018) 128:1–17. doi: 10.1016/j.phrs.2017.12.021 29284153

[158] Roskoski R . Properties of FDA-approved small molecule protein kinase inhibitors: A 2023 update. Pharmacol Res. (2023) 187:106552. doi: 10.1016/j.phrs.2022.106552 36403719

[159] Li AY McCusker MG Russo A Scilla KA Gittens A Arensmeyer K . RET fusions in solid tumors. Cancer Treat Rev. (2019) 81:101911. doi: 10.1016/j.ctrv.2019.101911 31715421

[160] Chen X Kang R Kroemer G Tang D . Broadening horizons: the role of ferroptosis in cancer. Nat Rev Clin Oncol. (2021) 18:280–96. doi: 10.1038/s41571-020-00462-0 33514910

[161] Lei G Zhang Y Koppula P Liu X Zhang J Lin SH . The role of ferroptosis in ionizing radiation-induced cell death and tumor suppression. Cell Res. (2020) 30:146–62. doi: 10.1038/s41422-019-0263-3 31949285 PMC7015061

[162] Wang L Huang H Li X Ouyang L Wei X Xie J . A review on the research progress of traditional Chinese medicine with anti-cancer effect targeting ferroptosis. Chin Med. (2023) 18:132. doi: 10.1186/s13020-023-00838-1 37833746 PMC10571466

[163] Oh S-J Ikeda M Ide T Hur KY Lee M-S . Mitochondrial event as an ultimate step in ferroptosis. Cell Death Discov. (2022) 8:414. doi: 10.1038/s41420-022-01199-8 36209144 PMC9547870

[164] Huang W Chen K Lu Y Zhang D Cheng Y Li L . ABCC5 facilitates the acquired resistance of sorafenib through the inhibition of SLC7A11-induced ferroptosis in hepatocellular carcinoma. Neoplasia. (2021) 23:1227–39. doi: 10.1016/j.neo.2021.11.002 34768109 PMC8591347

[165] Hu X He Y Han Z Liu W Liu D Zhang X . PNO1 inhibits autophagy-mediated ferroptosis by GSH metabolic reprogramming in hepatocellular carcinoma. Cell Death Dis. (2022) 13:1010. doi: 10.1038/s41419-022-05448-7 36446769 PMC9709074

[166] Yuan H Li X Yu M Cao Y Wu L Shi S . Injectable SF-platform orchestrates GPX4-targeted ferroptosis-autophagy-immunogenic circuit for overcoming oxidative resistance in triple-negative breast cancer. Theranostics. (2025) 15:8757–78. doi: 10.7150/thno.116013 40963899 PMC12439161

[167] Ma M-Z Chen G Wang P Lu W-H Zhu C-F Song M . Xc^−^ inhibitor sulfasalazine sensitizes colorectal cancer to cisplatin by a GSH-dependent mechanism. Cancer Lett. (2015) 368:88–96. doi: 10.1016/j.canlet.2015.07.031 26254540

[168] Gout PW Buckley AR Simms CR Bruchovsky N . Sulfasalazine, a potent suppressor of lymphoma growth by inhibition of the x(c)- cystine transporter: a new action for an old drug. Leukemia. (2001) 15:1633–40. doi: 10.1038/sj.leu.2402238 11587223

[169] Jadiya S Upmanyu N Sathiyanarayanan A Jain V Dubey R Buwade P . Formulation and development of novel sulfasalazine bilayer tablets for the treatment of arthritis associated with IBD: In-vitro and in-vivo investigations. J Pharm Sci. (2024) 113:1919–26. doi: 10.1016/j.xphs.2024.02.019 38401631

[170] Xie B Zhao H Ding Y-F Wang Z Gao C Li S . Supramolecularly engineered conjugate of bacteria and cell membrane-coated magnetic nanoparticles for enhanced ferroptosis and immunotherapy of tumors. Adv Sci (Weinh). (2023) 10:e2304407. doi: 10.1002/advs.202304407 37850572 PMC10700203

[171] Long Y Xu Z Yu J Hu X Xie Y Duan X . Targeting xCT with sulfasalazine suppresses triple-negative breast cancer growth via inducing autophagy and coordinating cell cycle and proliferation. Anticancer Drugs. (2024) 35:830–43. doi: 10.1097/CAD.0000000000001630 39016262

[172] Yang J Zhou Y Xie S Wang J Li Z Chen L . Metformin induces ferroptosis by inhibiting UFMylation of SLC7A11 in breast cancer. J Exp Clin Cancer Res. (2021) 40:206. doi: 10.1186/s13046-021-02012-7 34162423 PMC8223374

[173] Yang Y Yee D . IGF-I regulates redox status in breast cancer cells by activating the amino acid transport molecule xC^−^. Cancer Res. (2014) 74:2295–305. doi: 10.1158/0008-5472.CAN-13-1803 24686172 PMC4006361

[174] Hu K Li K Lv J Feng J Chen J Wu H . Suppression of the SLC7A11/glutathione axis causes synthetic lethality in KRAS-mutant lung adenocarcinoma. J Clin Invest. (2020) 130:1752–66. doi: 10.1172/JCI124049 31874110 PMC7108883

[175] Zhao T Chen X Wu J Chen S Gan Y Liu C . YY1-induced USP43 drives ferroptosis suppression by FASN stabilization and subsequent activation of SLC7A11 in ovarian cancer. Cell Death Dis. (2025) 16:589. doi: 10.1038/s41419-025-07886-5 40890129 PMC12402158

[176] Zhang J Liu Z Zhao W Li C Liu F Wang J . Targeting KRAS sensitizes ferroptosis by coordinately regulating the TCA cycle and Nrf2-SLC7A11-GPX4 signaling in hepatocellular carcinoma. Smart Med. (2025) 4:e70005. doi: 10.1002/smmd.70005 40390765 PMC12087391

[177] Saini KK Chaturvedi P Sinha A Singh MP Khan MA Verma A . Loss of PERK function promotes ferroptosis by downregulating SLC7A11 (System Xc^−^) in colorectal cancer. Redox Biol. (2023) 65:102833. doi: 10.1016/j.redox.2023.102833 37536085 PMC10412847

[178] Li Z Meng C Shen G Shan Y Wang J Abudukeremu D . Mitochondrial calcium uniporter drives chemoresistance in pancreatic cancer via glutathione-mediated stemness maintenance. Adv Sci (Weinh). (2026) 13:e07346. doi: 10.1002/advs.202507346 41560687 PMC13042756

[179] Kang L Wang D Shen T Liu X Dai B Zhou D . PDIA4 confers resistance to ferroptosis via induction of ATF4/SLC7A11 in renal cell carcinoma. Cell Death Dis. (2023) 14:193. doi: 10.1038/s41419-023-05719-x 36906674 PMC10008556

[180] Duan W-L Wang X-J Guo A Gu L-H Sheng Z-M Luo H . miR-141-3p promotes paclitaxel resistance by attenuating ferroptosis via the Keap1-Nrf2 signaling pathway in breast cancer. J Cancer. (2024) 15:5622–35. doi: 10.7150/jca.96608 39308683 PMC11414605

[181] He T Zhou F Su A Zhang Y Xing Z Mi L . Brusatol: A potential sensitizing agent for cancer therapy from Brucea javanica. BioMed Pharmacother. (2023) 158:114134. doi: 10.1016/j.biopha.2022.114134 36525821

[182] Cai SJ Liu Y Han S Yang C . Brusatol, an NRF2 inhibitor for future cancer therapeutic. Cell Biosci. (2019) 9:45. doi: 10.1186/s13578-019-0309-8 31183074 PMC6554866

[183] Ren D Villeneuve NF Jiang T Wu T Lau A Toppin HA . Brusatol enhances the efficacy of chemotherapy by inhibiting the Nrf2-mediated defense mechanism. Proc Natl Acad Sci USA. (2011) 108:1433–8. doi: 10.1073/pnas.1014275108 21205897 PMC3029730

[184] Yu X He Z Wang Z Ke S Wang H Wang Q . Brusatol hinders the progression of bladder cancer by Chac1/Nrf2/SLC7A11 pathway. Exp Cell Res. (2024) 438:114053. doi: 10.1016/j.yexcr.2024.114053 38663476

[185] Beeraka NM Zhang J Zhao D Liu J A U C Vikram Pr H . Combinatorial implications of Nrf2 inhibitors with FN3K inhibitor: *In vitro* breast cancer study. Curr Pharm Des. (2023) 29:2408–25. doi: 10.2174/0113816128261466231011114600 37861038

[186] Tian Z Yang Y Wu H Chen Y Jia H Zhu L . The Nrf2 inhibitor brusatol synergistically enhances the cytotoxic effect of lapatinib in HER2-positive cancers. Heliyon. (2022) 8:e10410. doi: 10.1016/j.heliyon.2022.e10410 36090218 PMC9449760

[187] Li J Zhang J Zhu Y Afolabi LO Chen L Feng X . Natural compounds, optimal combination of brusatol and polydatin promote anti-tumor effect in breast cancer by targeting Nrf2 signaling pathway. Int J Mol Sci. (2023) 24:8265. doi: 10.3390/ijms24098265 37175972 PMC10179160

[188] Yang Y Tian Z Guo R Ren F . Nrf2 inhibitor, brusatol in combination with trastuzumab exerts synergistic antitumor activity in HER2-positive cancers by inhibiting Nrf2/HO-1 and HER2-AKT/ERK1/2 pathways. Oxid Med Cell Longev. (2020) 2020:9867595. doi: 10.1155/2020/9867595 32765809 PMC7387975

[189] Wu T Harder BG Wong PK Lang JE Zhang DD . Oxidative stress, mammospheres and Nrf2-new implication for breast cancer therapy? Mol Carcinog. (2015) 54:1494–502. doi: 10.1002/mc.22202 25154499 PMC4697962

[190] TeSlaa T Ralser M Fan J Rabinowitz JD . The pentose phosphate pathway in health and disease. Nat Metab. (2023) 5:1275–89. doi: 10.1038/s42255-023-00863-2 37612403 PMC11251397

[191] Zhen X Zhang M Hao S Sun J . Glucose-6-phosphate dehydrogenase and transketolase: Key factors in breast cancer progression and therapy. BioMed Pharmacother. (2024) 176:116935. doi: 10.1016/j.biopha.2024.116935 38876050

[192] Meng Q Zhang Y Sun H Yang X Hao S Liu B . Human papillomavirus-16 E6 activates the pentose phosphate pathway to promote cervical cancer cell proliferation by inhibiting G6PD lactylation. Redox Biol. (2024) 71:103108. doi: 10.1016/j.redox.2024.103108 38457903 PMC10937312

[193] Li Y Zheng F Zhang Y Lin Z Yang J Han X . Targeting glucose-6-phosphate dehydrogenase by 6-AN induces ROS-mediated autophagic cell death in breast cancer. FEBS J. (2023) 290:763–79. doi: 10.1111/febs.16614 36048131 PMC10087799

[194] Li Y Han X Lin Z Wang C Fu Z Sun Q . G6PD activation in TNBC cells induces macrophage recruitment and M2 polarization to promote tumor progression. Cell Mol Life Sci. (2023) 80:165. doi: 10.1007/s00018-023-04810-y 37237244 PMC11073185

[195] Qian S Pan L Chen G Qiu F Ma Y Li R . Disulfidptosis: A novel cell death mechanism with pathological significance and therapeutic potential in diseases. Pharmacol Rev. (2026) 78:100127. doi: 10.1016/j.pharmr.2026.100127 41818911

[196] Li D Ma S . Beyond biochemical cascades: the biophysical execution of disulfidptosis via actin network collapse. Mol Biol Rep. (2026) 53:1239. doi: 10.1007/s11033-026-12413-6 42484924

[197] Song Z Yao Q Huang L Cui D Xie J Wu L . Glucose deprivation-induced disulfidptosis via the SLC7A11-INF2 axis: Pan-cancer prognostic exploration and therapeutic validation. Adv Sci (Weinh). (2025) 12:e08556. doi: 10.1002/advs.202408556 40709550 PMC12533382

[198] Wang C Zhou J Jin B Li G Zhang L Tian X . Disulfidptosis: molecular mechanisms and therapeutic targets. Signal Transduct Target Ther. (2026) 11:379. doi: 10.1038/s41392-026-03001-y 42711333 PMC13554092

[199] Tilekar K Upadhyay N Iancu CV Pokrovsky V Choe J-Y Ramaa CS . Power of two: combination of therapeutic approaches involving glucose transporter (GLUT) inhibitors to combat cancer. Biochim Biophys Acta Rev Cancer. (2020) 1874:188457. doi: 10.1016/j.bbcan.2020.188457 33096154 PMC7704680

[200] Wang Y Sun Z Zhao Z Pang J Chen J . Recent progress in the development of glucose transporter (GLUT) inhibitors. J Med Chem. (2025) 68:1033–50. doi: 10.1021/acs.jmedchem.4c02717 39746141

[201] Ren Z Zhao J Li S Yuan H . Targeting glucose transporter 1 (GLUT1) in cancer: Molecular mechanisms and nanomedicine applications. Int J Nanomedicine. (2025) 20:11859–79. doi: 10.2147/IJN.S534976 41036223 PMC12481445

[202] Zheng P Zhou C Ding Y Duan S . Disulfidptosis: a new target for metabolic cancer therapy. J Exp Clin Cancer Res. (2023) 42:103. doi: 10.1186/s13046-023-02675-4 37101248 PMC10134647

[203] Tay JY Ho JX Cheo FF Iqbal J . The tumour microenvironment and epigenetic regulation in BRCA1 pathogenic variant-associated breast cancers. Cancers (Basel). (2024) 16:3910. doi: 10.3390/cancers16233910 39682099 PMC11639800

[204] Saponaro C Malfettone A Ranieri G Danza K Simone G Paradiso A . VEGF, HIF-1α expression and MVD as an angiogenic network in familial breast cancer. PLoS One. (2013) 8:e53070. doi: 10.1371/journal.pone.0053070 23326384 PMC3543407

[205] Xu J Sun Y Huang B Zhang Y Shen H Zhou Z . A glucose metabolism-targeted nanoplatform amplifies disulfidptosis-associated immunogenic cell death in triple-negative breast cancer. Redox Biol. (2026) 96:104358. doi: 10.1016/j.redox.2026.104358 42660058 PMC13544341

[206] Lai Z-H Wang Y Li N-N Zeng L Liu R Li X . YTHDC1 orchestrates glucose and glutamate rewiring to overcome lethal metabolic stress in triple-negative breast cancer. Adv Sci (Weinh). (2026):e77492. doi: 10.1002/advs.77492 42669610 PMC13526702

[207] Liang Y Lan H Li Q Gao M Liu M Xu Z . Exploiting metabolic vulnerabilities through synergistic ferroptosis and disulfidptosis for breast cancer therapy. J Adv Res. (2026) 79:905–16. doi: 10.1016/j.jare.2025.03.052 40164329 PMC12766248

[208] Tolbatov I Marrone A . Disulfidptosis vs. ferroptosis: A comprehensive review of SLC7A11-mediated metal dyshomeostasis and cell death. Biomolecules. (2026) 16:671. doi: 10.3390/biom16050671 42194021 PMC13205066

[209] Hadian K Stockwell BR . The therapeutic potential of targeting regulated non-apoptotic cell death. Nat Rev Drug Discov. (2023) 22:723–42. doi: 10.1038/s41573-023-00749-8 37550363

[210] Boroń K Panfil A Sirek T Sirek A Zmarzły N Wróbel M . Subtype-consistent upregulation of ferroptosis-associated pathways in breast cancer with heterogeneous prognostic implications and systemic response to cryoablation. Int J Mol Sci. (2026) 27:3446. doi: 10.3390/ijms27083446 42074090 PMC13115818

[211] Iglesia MD Jayasinghe RG Chen S Terekhanova NV Herndon JM Storrs E . Differential chromatin accessibility and transcriptional dynamics define breast cancer subtypes and their lineages. Nat Cancer. (2024) 5:1713–36. doi: 10.1038/s43018-024-00773-6 39478117 PMC11584403

[212] Caputo A Vipparthi K Bazeley P Downs-Kelly E McIntire P Duckworth LA . Spatial transcriptomics suggests that alterations occur in the preneoplastic breast microenvironment of BRCA1/2 mutation carriers. Mol Cancer Res. (2024) 22:169–80. doi: 10.1158/1541-7786.MCR-23-0489 37878345 PMC10872731

[213] Zhu Q Balasubramanian A Asirvatham JR Chatterjee M Piyarathna B Kaur J . Integrative spatial omics reveals distinct tumor-promoting multicellular niches and immunosuppressive mechanisms in Black American and White American patients with TNBC. Nat Commun. (2025) 16:6584. doi: 10.1038/s41467-025-61034-3 40675986 PMC12271405

[214] Che L Zhu L Zhou L Zhou Y . Deciphering sorafenib resistance in hepatocellular carcinoma via ferroptotic mechanisms. Biochim Biophys Acta Rev Cancer. (2026) 1881:189566. doi: 10.1016/j.bbcan.2026.189566 41763496

[215] Han C Ge M Xing P Xia T Zhang C Ma K . Cystine deprivation triggers CD36-mediated ferroptosis and dysfunction of tumor infiltrating CD8+ T cells. Cell Death Dis. (2024) 15:145. doi: 10.1038/s41419-024-06503-1 38360744 PMC10869360

[216] Wan J Shi J-H Shi M Huang H Zhang Z Li W . Lactate dehydrogenase B facilitates disulfidptosis and exhaustion of tumour-infiltrating CD8+ T cells. Nat Cell Biol. (2025) 27:972–82. doi: 10.1038/s41556-025-01673-2 40461882

[217] Lang X Green MD Wang W Yu J Choi JE Jiang L . Radiotherapy and immunotherapy promote tumoral lipid oxidation and ferroptosis via synergistic repression of SLC7A11. Cancer Discov. (2019) 9:1673–85. doi: 10.1158/2159-8290.CD-19-0338 31554642 PMC6891128

[218] Li F Qi Q Qiao Y Huang Y Lu Y Gu K . Curcumenol inhibits malignant progression and promotes ferroptosis via the SLC7A11/NF-κB/TGF-β pathway in triple-negative breast cancer. Int J Mol Med. (2025) 56:111. doi: 10.3892/ijmm.2025.5552 40377003 PMC12121984

